# Somato-dendritic decoupling as a novel mechanism for protracted cortical maturation

**DOI:** 10.1186/s12915-016-0270-5

**Published:** 2016-06-21

**Authors:** Taylor Chomiak, Johanna Hung, Minh Dang Nguyen, Bin Hu

**Affiliations:** Division of Translational Neuroscience, Department of Clinical Neurosciences, Hotchkiss Brain Institute, Cumming School of Medicine, University of Calgary, 3330 Hospital Drive NW, Calgary, Alberta T2N 4N1 Canada

**Keywords:** Pyramidal, Neuron, Neocortex, Excitability, Remote, Memory

## Abstract

**Background:**

Both human and animal data indicate that disruption of the endogenously slow maturation of temporal association cortical (TeA) networks is associated with abnormal higher order cognitive development. However, the neuronal mechanisms underlying the endogenous maturation delay of the TeA are poorly understood.

**Results:**

Here we report a novel form of developmental plasticity that is present in the TeA. It was found that deep layer TeA neurons, but not hippocampal or primary visual neurons, exist in a protracted ’embryonic-like’ state through a mechanism involving reduced somato-dendritic communication and a non-excitable somatic membrane. This mechanism of neural inactivity is present in intact tissue and shows a remarkable transition into an active somato-dendritically coupled state. The quantity of decoupled cells diminishes in a protracted and age-dependent manner, continuing into adolescence.

**Conclusions:**

Based on our data, we propose a model of neural plasticity through which protracted compartmentalization and decoupling in somato-dendritic signalling plays a key role in controlling how excitable neurons are incorporated into recurrent cortical networks independent of neurogenesis.

**Electronic supplementary material:**

The online version of this article (doi:10.1186/s12915-016-0270-5) contains supplementary material, which is available to authorized users.

## Background

Delayed functional maturation of the temporal association cortex (TeA) during early postnatal development is a well-recognized phenomenon of brain maturation that is conserved across species [1–5]. Cognitive neuroscientists have long realized that it is during this critical window that large-scale developmental changes in the brain co-occur with marked changes and expansion in cognitive capabilities that seem to follow a defined hierarchical chronological pattern [6–10]. This pattern of development is thought to allow more specialized higher order information processing streams to incrementally emerge, but only after lower level neural afferents from primary cortices become more mature and stable [9, 11–13].

Largely derived from early maturing brain circuits, popular neurobiological models of brain development emphasize that the intrinsic neural processing capabilities of cortical networks develop synchronously [8, 14] and are present very early during development to facilitate activity-dependent refinement of circuits through selective elimination of initial exuberant growth [7, 15, 16]. However, a major challenge for this type of developmental model is that it is difficult to reconcile with the findings of different, and often opposing, trends of protracted maturation trajectories, both between cortical regions and within high-order recurrent cortical networks (e.g. TeA versus prefrontal cortex) [13, 17, 18]. Furthermore, during the postnatal period, cognitive functional development is often associated with a protracted increase in activation areas and responses along the more specialized information processing streams in the TeA [2, 3, 5, 10, 19]. This supports both theoretical and empirical findings that limiting initial computational resources may actually facilitate, rather than hinder, normal cognitive development in recurrent neuronal networks [6, 7]. These seemingly contradictory findings, therefore, raise questions regarding the possible neurobiological mechanism of protracted high-order cortical maturation.

The neuronal mechanisms underlying delayed maturation of the TeA are poorly understood. Previous models of early postnatal maturation have often focused on structural constraints such as synaptic and/or dendritic complexity [7, 8, 15]. However, there is accumulating evidence that developmentally regulated functional constraints may also be important. For instance, electrophysiological recordings and functional imaging have shown that the TeA lacks significant excitability and functional connectivity during the first month of postnatal life [1, 20], and accelerating TeA maturation during early postnatal development can disrupt normal behavioural development [20, 21]. These observations are also in line with data consistently reporting that electrical activity is not required for the establishment of basic neuronal morphology [22–24]. Together, these findings suggest that neurobiological processes that support independently regulated neuronal morphological and electrophysiological maturation may provide an important additional mechanism of protracted functional cortical maturation.

## Results

### Reduced TeA excitability despite continued growth

**Fig. 1:**
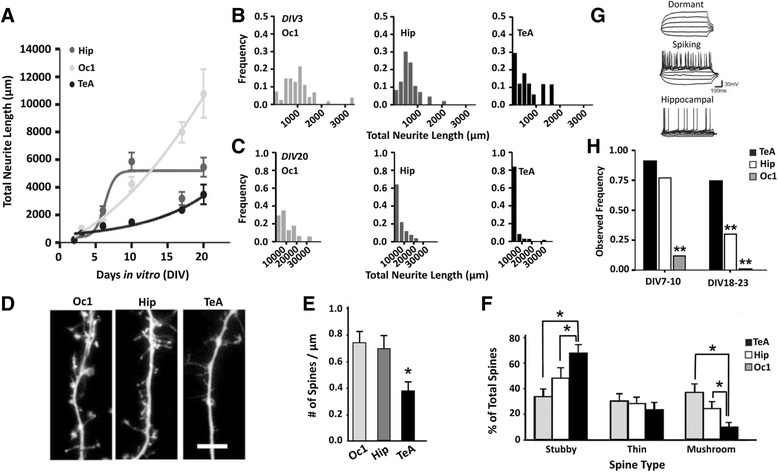
Reduced TeA excitability despite continued growth. **a** Neurons of various ages were transfected with an eGFP plasmid for morphological analysis (Oc1, *n* = 214; Hip, *n* = 213; TeA, *n* = 289). **b**–**c** Histograms showing neuron size distribution in Oc1, Hip, and TeA at DIV3 **b** and DIV20 **c**. The growth time course varied by culture type as a two-way ANOVA revealed a significant main effect of culture type (F_(2,698)_=37.45, *p* < 0.0001; Two-way ANOVA) and culture age (F_(5,698)_=68.21, *p* < 0.0001; Two-way ANOVA) on total neurite length. There was also a significant interaction between culture type and age (F_(10,698)_=11.39, *p* < 0.0001; Two-way ANOVA). **d** Images of GFP-labelled spines at 100× magnification from DIV20 cultures. Scale bar = 5 μm. **e** Summarized data for spine density at DIV20. The total dendrite length used for spine densitometry: TeA = 1803 μm; Hip = 1038 μm; Oc1 = 840 μm (Hip, *n* = 10; TeA, *n* = 11; Oc1, *n* = 6). **f** The percentage of stubby (*F*_(2,84)_ = 21.19; *p* < 0.0001; one-way ANOVA), thin (*F*_(2,84)_ = 1.55; *p* = 0.22; one-way ANOVA), and mushroom-shaped (*F*_(2,84)_ = 29.34; *p* < 0.0001; one-way ANOVA) spines per neurite for different regions. * *p* < 0.05. **g** Voltage recordings obtained under current-clamp from a TeA dormant pyramidal cell (*top*), a TeA spiking pyramidal cell (*middle*), and a hippocampal spiking pyramidal cell for comparison (*bottom*) during depolarization. Current pulses were injected from a holding or resting potential of around –60 to –70 mV. Detailed electrophysiological characterization revealed input resistance (5.01 ± 1.1 GΩ *n* = 9 vs. 0.30 ± 0.08 GΩ *n* = 10; *t*_(9)_ = 4.12 Welch-corrected *p* = 0.003) and capacitance (4.1 ± 0.3 pF *n* = 9 vs. 105.2 ± 21.8 pF *n* = 10; t_(9)_ = 4.64 Welch-corrected *p* = 0.001) between phenotypes are significantly different. Note that given the order of magnitude difference in input resistance between dormant (GΩ) and spiking (MΩ) cells, there was also order of magnitude difference in current step amplitude (e.g. 5-pA steps vs. 50-pA steps). Traces are representative of recordings during the TeA growth phase (DIV10–23). **h** Significantly more TeA pyramidal cells are dormant. DIV7–10; Oc1 significantly lower than both TeA and Hip *p* < 0.0001. DIV18–23; Oc1 and Hip significantly lower than TeA *p* < 0.0001 and *p* = 0.0019 respectively. *p* values determined by a Fisher’s exact test on observed phenotype frequency. *p < 0.05; **p < 0.01

We first wanted to evaluate the single-cell morphological developmental trajectory of the TeA compared to other brain regions over the first several weeks of postnatal neuronal development that corresponds to the period of reduced TeA functional connectivity [1]. Given that neurite outgrowth is commonly used as an index of neural maturation [25], we first evaluated the developmental trajectory of TeA neurite growth. To this end, TeA tissue from the cortical plate was harvested at P0 and grown in primary culture as this allows us to easily isolate cells destined to be neocortical pyramidal neurons [26]. Hippocampal and primary visual cortex (Oc1) cultures were prepared under identical conditions to serve as positive controls, and cells were transfected with green fluorescent protein (GFP) for morphological quantification. We found that, unlike the hippocampus and Oc1, a distinctly slower, but nevertheless increasing, neurite growth trajectory was observed in the TeA (Fig. 1a–c). We also evaluated dendritic spine density from GFP transfected cells (Fig. 1d–f). Dendritic spines are small protrusions that typically receive excitatory inputs, and their morphology is correlated with the strength and maturity of the synapse that they host [27]. Not only did we find that the TeA had significantly lower spine density at DIV20 in general (Fig. 1e), but also that a significantly lower percentage of these were of the larger more mature ‘mushroom’ type variety (Fig. 1f). Given that action potentials (APs) can influence dendritic growth and dendritic spine density [28], we also decided to evaluate the ability of TeA neurons to fire action potentials by targeting whole-cell patch-clamp recordings to visually identified pyramidal neurons as routinely done under light microscopy [29]. Interestingly, when recordings were made from the TeA, they revealed two distinct functional phenotypes of pyramidal cells despite their similar appearance: a spiking phenotype and a non-spiking embryonic-like phenotype. Spiking cells typically discharged regenerative over-shooting APs during depolarization (Fig. 1g, middle) and showed characteristic electrophysiological properties of maturing pyramidal neurons. By contrast and similar to findings previously reported for very immature neural phenotypes [30–33], non-spiking pyramidal cells during prolonged and large (e.g. up to 0 mV) membrane depolarization failed to discharge APs and also lacked the outward rectifying current characteristic of spiking cells (Fig. 1g, top; also see Additional file 1: Figure S1). We have defined these non-spiking cells as ’dormant’, and ruled out the possibility that this phenotype reflects a failure to obtain a whole-cell recording configuration (see Methods; Additional control experiments section).

**Fig. 2:**
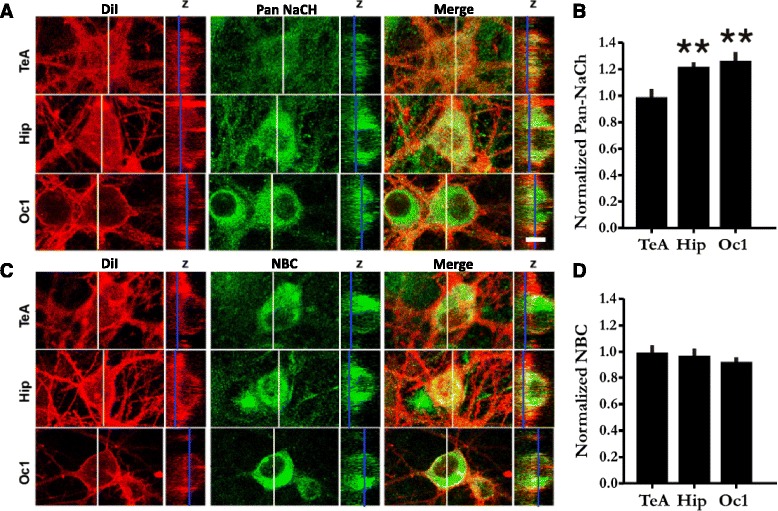
Selective reduction of sodium channel α subunit expression in TeA neurons. **a** Confocal micrographs of TeA, hippocampal, and Oc1 neurons after immunolabelling with antibodies against the α-sodium channel subunit (Pan NaCH, *green*). Neurons were co-stained with DiI (*red*) to delineate the plasma membranes and NeuN (not shown) to verify their identity as neurons. *White vertical lines* on the XY image indicate the orthogonal plane where the Z-sections were taken. *Blue vertical lines* indicate the Z-location of the XY plane shown. In each group, cultures were processed for immunocytochemistry and DiI labelling completely in parallel, and images were captured under identical conditions. Scale bar = 10 μm. ** *p* < 0.01. **b** Quantification of peri-plasmalemmal channel expression (see Methods). *n* = 28, *n* = 33 and *n* = 19 for TeA, hippocampal and primary visual respectively. *F*_(2,77)_ = 12.41 *p* < 0.0001, ANOVA and Tukey’s post hoc test (TeA significantly different than Oc1 and Hipp (*p* < 0.001). Hipp and Oc1 *p* > 0.05). **c** Confocal micrographs of TeA, hippocampal and Oc1 neurons after immunolabelling with antibodies against the sodium bicarbonate co-transporter (NBC, *green*). **d** Quantification of peri-plasmalemmal NBC expression. *n* = 27, *n* = 18 and *n* = 10 for TeA, hippocampal and primary visual respectively. There is no significant difference; *F*_(2,52)_ = 0.76, *p* = 0.47. Cultures were also processed for immunocytochemistry and DiI labelling completely in parallel and images were captured under identical conditions

Next we obtained recordings from other brain regions to see if this phenotype was a general feature of neural maturation. Unlike the TeA, however, when recordings were targeted to pyramidal cells cultured under identical conditions from the hippocampus or Oc1, significantly more of them exhibited a spiking phenotype, particularly at older ages (see Fig. 1g, bottom, for example and Fig. 1h; DIV7–10: TeA 91 % *n* = 22, Hip 77 % *n* = 26, primary visual 12 % *n* = 17 and DIV18–23: TeA 74 % *n* = 35, Hip 30 % *n* = 20, primary visual 0 % *n* = 10). In addition, we found that TeA neurons expressed significantly less type 1 sodium channel α subunits (NaCH) that are preferentially expressed in the cortex and soma in the rat [34] (Fig. 2a–b). This reduction was, however, not due to a generalized reduction in protein expression as there was no significant difference in the sodium bicarbonate co-transporter (NBC) (Fig. 2c–d), a common membrane protein involved in ion homeostasis [35].

### Neuronal dormancy is associated with poor somato-dendritic coupling and a specific lipophilic cytosolic structural organization

The fact that dendritic electrophysiological maturation can precede that of the soma [36] and that dendritic surface area is several-fold greater than that of the soma [37] indicates that the majority of somatically recorded current reflects dendritic conductances. Indeed, previous experimental evidence has directly confirmed this [38] and is consistent with the fact that hyperpolarizing-activating cationic current (*I*_h_), which is commonly observed in somatic recordings [30], is not present in the soma but increases in density with increasing distance from the soma [39]. Therefore, one possibility may be that there is a complete and uniform lack of functional conductances in TeA dendrites. While unlikely, we nevertheless tested this by applying the same recording procedure directly to dendrites (Additional file 2: Figure S2). Although TeA dendrites are generally quite delicate and difficult to record from, when dendritic recordings were obtained and in contrast to somatic recordings, they indeed had significantly greater membrane conductance at both hyperpolarized (–100 mV) and, in particular, depolarized (+50 mV) potentials even in the same neuron (Additional file 2: Figure S2). Moreover, all dendritic recordings exhibited outward rectification (Additional file 2: Figure S2) and, in at least 50 % of dendritic recordings, inward spike currents with peak amplitudes ranging from 0.24–2.9 nA (mean = 1.7 ± 0.8 nA; not shown) were also evident with step depolarization. Thus, it does not appear that the dormant phenotype results from a general lack of membrane conductances (also see below).

A second possibility may be that the soma of the dormant phenotype is functionally compartmentalized. As a functionally isolated soma would fail to incorporate the dendritic capacitance load [40], we first measured capacitance values of dormant and spiking neurons. Indeed, we found that dormant neurons consistently exhibited a significantly smaller membrane capacitance than that of spiking cells (Additional file 3: Figure S3), similar to what was previously observed in immature neurons [30–33]. In fact, as the recorded membrane capacitance of dormant neurons is too large to represent a cell-attached configuration [41] and too small to incorporate the dendritic compartment [40], we calculated the expected somatic membrane capacitance based on dimensions of the soma. As predicted, the calculated somatic values correspond to the experimentally measured capacitance values of the dormant phenotype (see Additional file 3: Figure S3).

**Fig. 3:**
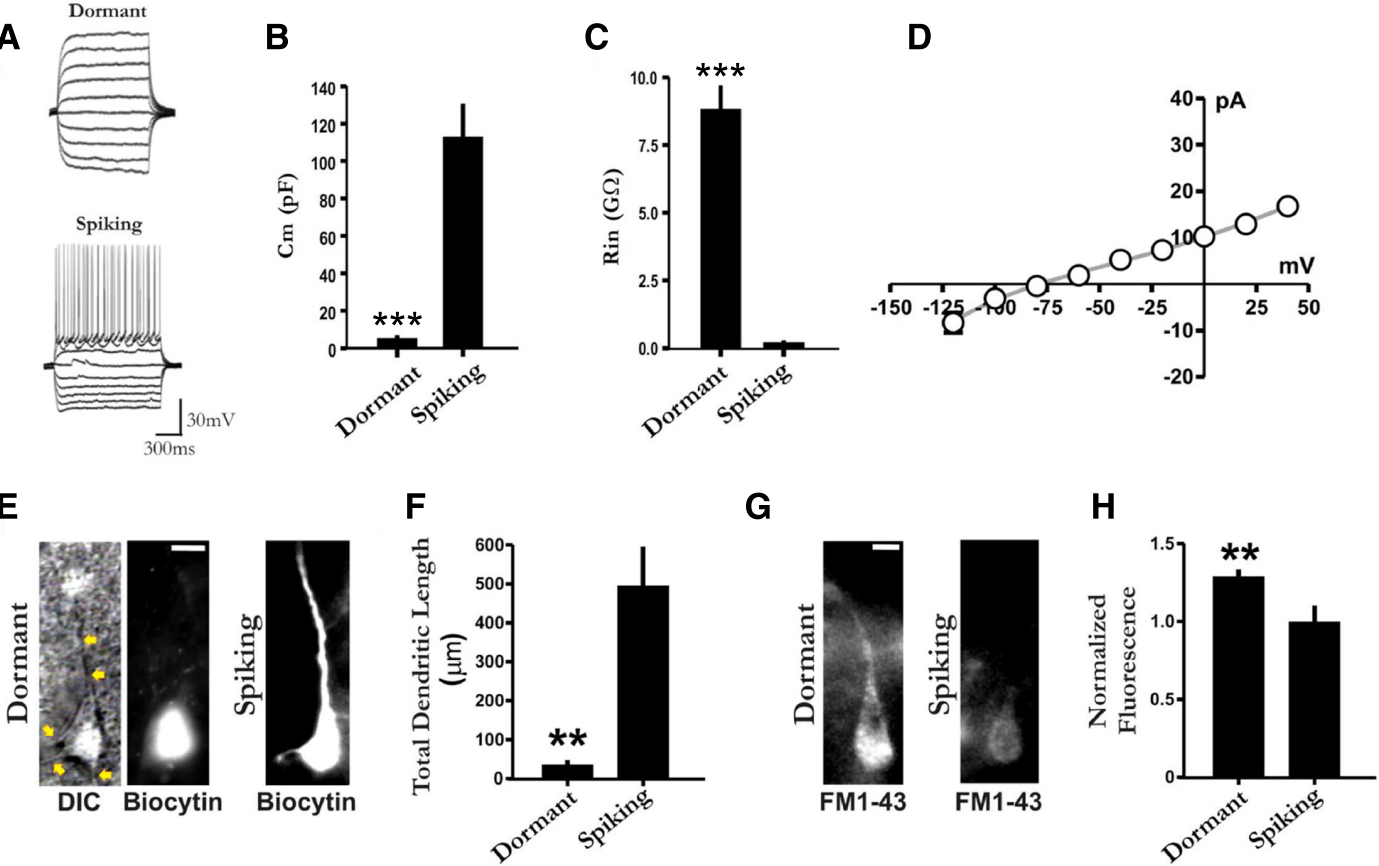
Dormant neurons exist in intact tissue and exhibit a specific cytosolic structural organization. **a** Typical in situ recordings obtained under current clamp from a dormant (*top*) and a spiking pyramidal cell (*bottom*). The former is characterized by the conspicuous absence of action potentials. Note in spiking cells, but not dormant cells, spontaneous synaptic activity is present. **b**–**c** Summarized data of membrane capacitance (*C*_m_; dormant *n* = 18, spiking *n* = 13; *t*_(12)_ = 5.56 Welch-corrected *p* < 0.001) and input resistance (*R*_in_; dormant *n* = 18, spiking *n* = 13; *t*_(17)_ = 9.99 Welch-corrected *p* < 0.001) between the two cell types in situ. **d** Similar to behaviour observed in vitro, under voltage-clamp (–60 mV) conditions, dormant neurons exhibited negligible current and a quasi-linear current-voltage relationship. **e** Representative images of a biocytin (0.5–1 %) labelled dormant (*left*) and spiking neuron (*right*), illustrating their notable difference in ability to label dendrites. Note that the lack of dendritic labelling is not due to the fact that dormant neurons lack dendrites, as dendrites could always be visualized under DIC microscopy (*left; yellow arrows*). Dormant DIC and biocytin images are of the same cell. **f** Summary data of the total dendritic length labelled between the two phenotypes (*t*_(5)_ = 4.42 Welch-corrected *p* = 0.0068; *n* = 7 and *n* = 6 respectively). **g** Representative images of both dormant (*left*) and spiking (*right*) neurons when the lipid-binding amphipathic dye FM1-43 (3–10 μM) was included in the pipette. **h** Summary data of the FM1-43 fluorescence signal between the two phenotypes (*t*_(19)_ = 3.30, *p* = 0.0037; *n* = 14 dormant and *n* = 7 spiking). **e** and **g** scale bars = 10 μm. ** *p* < 0.01; ***p < 0.001

To further investigate this issue under more physiological conditions and to rule out the possibility that this phenotype may be an artefact of the culture conditions, we obtained whole-cell recordings from visually identified pyramidal neurons in acute brain slices. In some experiments, dormant cell neuronal identity was confirmed by co-staining with the neuron-specific marker NeuN (Additional file 4: Figure S4). Importantly, dormant cells were also observed in intact tissue and had virtually identical electrophysiological properties to those in vitro (Fig. 3a–d). In addition and similar to the results in vitro, small membrane capacitance values, roughly 20-fold less than that of the spiking phenotype, were also associated with the dormant phenotype in intact tissue (Fig. 3b). In further support of this electrophysiological finding that the soma of dormant neurons is isolated from the dendrites, we also tested to see if the diffusible aqueous dye biocytin would remain localized in the soma of dormant but not spiking neurons. Indeed, the soma of both dormant and spiking types could be identified via biocytin labelling (Fig. 3e), but unlike spiking cells, dormant cells generally only had a dye-labelled soma and lacked dendritic biocytin labelling despite the fact that dendrites could be visualized under DIC microscopy (Fig. 3e–f).

Given that a greater degree of intracellular membrane biogenesis is needed during neuronal maturation [42], we also tested to see if there was a difference in the spectral density of intracellular binding of the lipid-binding dye FM1-43 between spiking and dormant cells. The rationale for this is that it may not only help explain previous reports that complete labelling of the somatic and dendritic compartments with aqueous dye can sometimes be more difficult in neurons from younger animals during intracellular recording [43, 44], but it may also provide insight into a potential mechanism contributing to somato-dendritic decoupling. To this end, the amphipathic dye FM1-43, which does not fluoresce when in an aqueous volume fraction, was delivered intracellularly through the patch pipette during recordings done to verify electrophysiological phenotype. It was found that dormant cells exhibited a significantly higher fluorescence spectral density signal than that of spiking cells (Fig. 3g–h), consistent with an intracellular somatic structure involving a densely ordered cytoplasmic space [45].

**Fig. 4:**
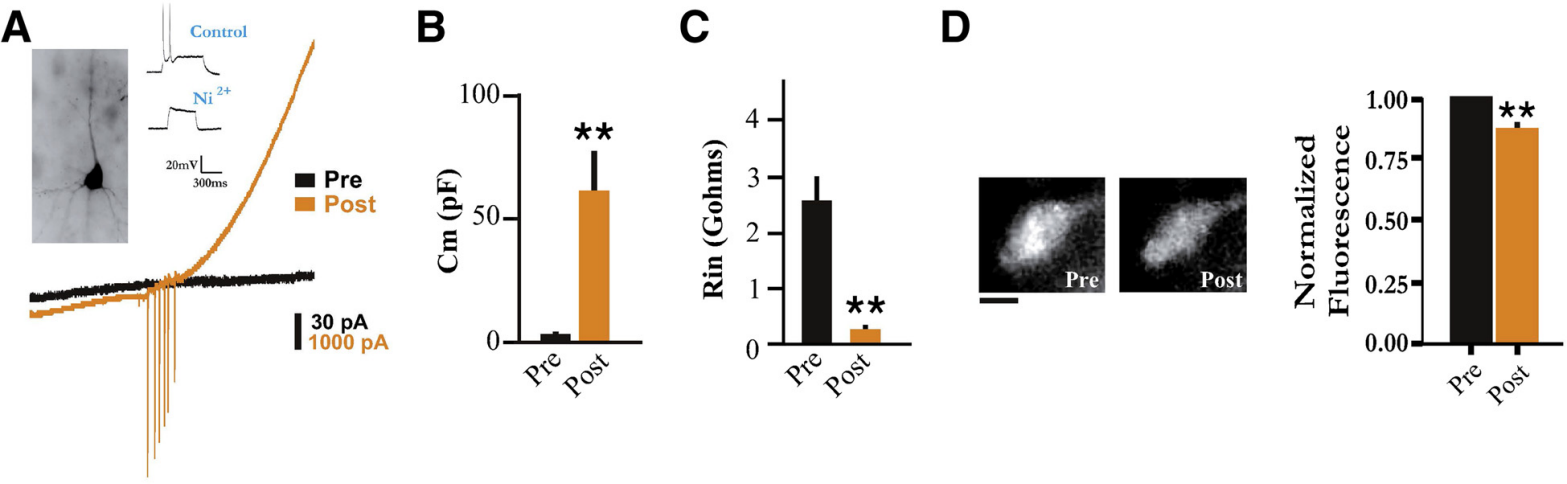
Disrupting the specific cytosolic structural organization of dormant neurons can unmask dendritic surface area and calcium spikes. **a** Current traces from the same neuron ’pre’ (*black*) and ’post’ (*orange*) micro-electrical stimulation. The cell was voltage-clamped at –60 mV and a voltage ramp from –120 mV to +60 mV was applied before and after stimulation. *Insets*; biocytin labelling of the same cell (*left*) and pharmacological blockage of somatically recorded spikes with the calcium channel blocker nickel from another cell (*right*). **b**–**c** Summary data of the membrane capacitance (*C*_m_ *t*_(10)_ = 3.56, *p* = 0.005, *n* = 11; paired *t* test) and input resistance (*R*_in_ *t*_(10)_ = 4.83, *p* = 0.001, *n* = 11; paired *t* test) from the same cells pre and post stimulation. **d** An example (*left*) and summary data (*right*; *t*_(12)_ = 5.73, *p* < 0.0001, *n* = 13; paired t test) of the FM1-43 fluorescence spectral density signal pre and post stimulation. As expected, there was also a significant decrease in access resistance associated with these fluorescence spectral density changes pre and post micro-electrical stimulation (37.5 ± 5.3 vs. 21.6 ± 9.4 MΩ; *t*_(10)_ = 5.10; *p* = 0.0005). Note that the access resistance was on average around 10 % of the input resistance, and was even lower for the decoupled state. Scale bar 5 μm. ** *p* < 0.01

Next we tested if local electrical micro-stimulation could disrupt somato-dendritic decoupling in the same recording. This would provide the most direct evidence of decoupling. The rationale for this is based on the idea that electrical force from applied voltages can induce rapid changes in intracellular lipid orientation and associated microtubule translocation [46, 47], and it has also been shown by Patel and Poo that electrical fields can be used to alter neuronal structure [48]. Indeed, it was found that following a sustained period (e.g. tens of seconds) of electrical micro-stimulation (see Methods), a phenotype switch was often observed characterized by increased capacitive load that was now accessible and accompanied by decreased input resistance, recordable spikes and outward rectification similar to that observed in dendritic recordings (Fig. 4a–c). Furthermore, and to provide evidence of somatic cytosolic reorganization, FM1-43 spectral density signals were also measured before and after stimulation. Consistent with a mechanism involving a densely ordered cytoplasmic space [45], there was a significant reduction in the fluorescence spectral density signal in these same cells following the appearance of somatically recorded currents (Fig. 4d). In an additional series of experiments we also found that the majority (80 %; *n* = 5) of these somatically recorded spikes observed following stimulation were abolished by the calcium channel blocker Ni^2+^ (1–5 mM; see Fig. 4a inset traces), further confirming that this phenotypic transition was associated with dendritic capacitive load coupling and Ca^2+^ spikes [49].

### TeA neuronal dormancy decreases with advancing age

**Fig. 5:**
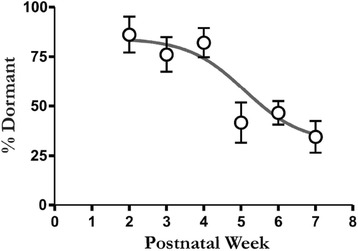
TeA age-dependent decrease in neuronal dormancy. Chronological changes in neuronal dormancy of the TeA are indexed by the proportional decrease in the dormant phenotype by postnatal week. Note the striking pre-pubescent transition that occurs between the fourth and fifth postnatal weeks. Neuronal dormancy was higher in juvenile animals (postnatal weeks 2–4; *n* = 23) and lower in mature animals (postnatal weeks 5–7; *n* = 34; *F*_(5,51)_ = 6.87, *p* < 0.0001, ANOVA; with week 7 significantly lower than weeks 2–4, Tukey’s post hoc test *p* ≤ 0.05). Data are from a total of 57 animals. Week 2 (animals/cells; 5/18), week 3 (10/43), week 4 (8/33), week 5 (3/14), week 6 (17/84) and week 7 (14/63)

Finally, if decoupled neurons participate in the process of age-dependent maturation in the TeA [1], then the phenomenon of neuronal dormancy itself should also exhibit an age-dependent modification. To this end, we evaluated the developmental trajectory of neuronal dormancy by systematically determining the percentage of dormant neurons in the TeA of rats at different ages (Fig. 5). Given the relatively small change in cell counts between juvenile and mature animals (Additional file 5: Figure S5), a large increase in the proportion of coupled neurons would indicate an endogenous and protracted shift towards a functionally mature network. Indeed, it was found that a large proportion (about 75 %) of recorded neurons were dormant during postnatal weeks 2–3 (Fig. 5). However, starting between the fourth and fifth postnatal weeks, a striking decrease in the proportion of dormant cells (i.e. increase in coupled cells) was observed (Fig. 5) that appears to persist into adolescence [50].

## Discussion

### Neural circuit decoupling and dormancy rather than neurogenesis

An intriguing possibility related to protracted functional maturation of the TeA network would be to produce new neocortical pyramidal neurons through the process of postnatal neurogenesis. Although the features of dormant neurons are reminiscent of very immature neurons found during adult neurogenesis [32], an important feature of the postnatal neocortex is that it does not appear to produce new neurons [51] (Additional file 5: Figure S5). In addition, although we did not specifically examine the chemical nature of dormant neurons, i.e. whether they are destined to an interneuron fate, the high proportion of this phenotype (about 75 %) during early postnatal development is inconsistent with the relatively few (<25 %) interneurons present in the neocortex [52]. Rather, our data indicate that somato-dendritic decoupling has led to a dormant phenotype and a previously under-appreciated maturational stage of pyramidal cells, an assertion supported by their morphology and the fact that these neurons also have sub-cortical projections [53].

### Physiological basis of somato-dendritic decoupling

Polarized neurons are highly compartmentalized and can maintain discrete and independent signalling mechanisms within restricted sub-cellular domains [54–56]. Many previous reports indicate that the somatic compartment of an immature neuron can possess few cationic conductances, especially when it is artificially isolated from all neurites [57] or at a developmental stage absent of complex dendritic structure [30–33]. On the other hand, axons and dendrites isolated from cell somata can still maintain RNA and protein synthesis, as well as membrane trafficking [54–56]. Indeed, dendritic and axonal growth can proceed at different rates and operate via different mechanisms [58], and somatic activity does not appear to be required for axonal development [59]. Consistent with these studies, decoupled TeA neurons appear to develop and retain basic dendritic morphology and long axonal projections [53], suggesting the presence of a unique, but not entirely unexpected, homeostatic state allowing cell maturation to be carried out in a highly parallel and compartmentalized fashion.

The significantly reduced somato-dendritic coupling in dormant cells is supported by previous studies [43, 44] as well as our own observations, indicating that neurons in younger animals can sometimes be more difficult to rapidly and extensively fill with intracellular aqueous (particularly Alexa), but not amphipathic dye. In coupled neurons, however, we have observed mature electrophysiological properties and individual pyramidal neuron firing patterns comparable to those reported in the literature [30, 60–62]. This suggests that the mechanism underlying somato-dendritic decoupling is likely modifiable under physiological conditions, especially when considering the fact that a whole range of physiological neuronal firing patterns can be produced by a two-compartmental computational model described by two parameters: dendritic size and somato-dendritic interface resistance [63]. In fact, the thin intracellular cytoplasmic space and complex internal protein-lipid structure characteristic of immature neurons may significantly increase interface resistance [42, 45, 64].

### Maintenance of somato-dendritic decoupling

Several regulatory mechanisms of neuronal maturation may influence the degree of somato-dendritic decoupling and viability of decoupled cells. For example, in many immature cell types, the early expression of functional calcium channels can give rise to calcium-mediated action potentials that are conserved during transient periods of development before transitioning into a more mature sodium-mediated action potential phenotype [28, 65]. In the rat cerebellum, activation of immature granule cells results in calcium-mediated action potentials during early (but not late) postnatal development [66], while in insects, the period with which calcium-mediated action potentials operate coincides with early dendritic remodelling of pupal motoneurons [67]. A multitude of developmental genomic and epigenetic programs are hosted in the soma, and their activation often requires external stimulation and calcium influx [16, 28]. The fact that in decoupled TeA neurons calcium- rather than sodium-mediated spikes can be recorded following electrically disrupted decoupling and the fact that sodium-mediated spikes are present in spiking neurons from more mature tissue (Additional file 6: Figure S6) further support this developmental model in the TeA. Decoupling may therefore be an important mechanism in regulating the accessibility of these signals on the somatic genomic/epigenomic machinery involved in cellular maturation.

A second mechanism that may contribute to the prolonged dormancy of TeA neurons may be related to the hypothesis that neuronal maturation in the mammalian cortex may be governed by regional specific cues [68–70] and/or the hierarchical pattern of afferent and efferent connectivity [8]. In this regard, the maturation speed of TeA neurons may be influenced by several factors, including the levels of afferent activity and trophic signalling. For instance, trophic signalling via brain-derived neurotrophic factor (BDNF) is important in nervous system maturation [71, 72]. It can stimulate dendritic growth [73] (also see Additional file 3: Figure S3) and induce somato-dendritic communication and transcriptional regulation [74], and it can up-regulate functional sodium channel expression [75]. Following BDNF signalling, positive feedback between voltage-gated and capacitive calcium current, as well as autocrine and paracrine trophic signalling [76, 77], may therefore allow for somato-dendritic coupling to emerge during postnatal dendritic remodelling and Golgi expansion [42].

Finally, the absence of significant somatic cationic channels may further serve a role in protecting decoupled cells from cationic channel-dependent apoptotic cell death and elimination [78]. However, the lack of significant somatic potassium conductance in the decoupled state does suggest an atypical ionic basis for the establishment of somatic resting membrane potential in the decoupled state. While chloride conductance and cationic co-transporters are crucial in ion homeostasis [79], they may also play a role in establishing resting membrane potential under conditions of reduced potassium conductance [80, 81].

## Conclusions

Somato-dendritic decoupling and neuronal dormancy represent a unique form of brain plasticity associated with cognitive network maturation in the TeA. Our data support the idea that, independent of neurogenesis, new functional units may be gradually added to the TeA cortical network with increasing proportions of somato-dendritically coupling spiking neurons. Whether a similar type of plasticity also exists in other cortical layers and/or in other association cortices remains unclear. Nevertheless, the polysensory TeA in rodents forms a large-scale recurrent neuronal network and shares many structural and functional similarities to that of primates, including various forms of memory and attention control [53, 82–86]. Pharmacological agents or pathological conditions that alter the maturational trajectory of the temporal lobe may therefore have a detrimental impact on cognitive development [87, 88]. Indeed, exposure to valproic acid (VPA), which significantly increases the risk of autism in children [89], not only stimulates TeA dendritic growth and reduces neuronal dormancy in rodents, but it can also lead to pathological features characteristic of autism [20, 21]. These findings, when taken together, indicate that decoupling and neural dormancy may play a very important role in normal cognitive development.

## Methods

### In vitro cultures

All experimental protocols were approved by the University of Calgary Conjoint Faculties Research Ethics Board (AC12-0239). Cells were prepared by a standard protocol. The dimensions and location of the cortical regions of the P0 rat were referenced from the *Atlas of the Developing Rat Brain* [90]. The entire hippocampus (Hip), primary visual cortex (Oc1) and the polymodal temporal association cortex (TeA), also known as area Te2 which is also part of the visual system [91, 92], were removed by microdissection and incubated in sterile filtered enzymatic solution containing CaCl_2_ (Sigma, Oakville, ON), L-cysteine (Sigma), EDTA and 20 U/ml papain (Worthington, Lakewood, NJ) at 37 °C for 30 min. All cells were then washed with fresh media and dissociated by triturating though a set of three flame-prepared Pasteur pipettes of decreasing calibre. Cells were counted with a hemocytometer and cultured on glass coverslips (previously coated with poly-D-lysine and laminin) and placed in a 24-well plate at a culture density of about 175 × 10^3^ cells/cm^2^. Cultures represent cells from multiple (up to 10) pups. The final volume of growth medium was maintained at 800 μl. One half of the media was replaced every 3–4 days. Culture media consisted of Basal Medium Eagle supplemented with glucose, B-27, penicillin-streptomycin, Na pyruvate, HEPES (Sigma), L-glutamine (Sigma), and 4 % fetal bovine serum (FBS). Recordings were obtained at room temperature (RT) from cells immersed in extracellular bath solution (EBS) containing (in millimolar units): 3.0 CaCl_2_, 2.0 MgCl_2_, 135 NaCl, 5.0 KCl, 10 glucose, 5.0 HEPES and a pH and osmolality of around 7.3 and 310 mOsm respectively. Cultures were fixed for 20 min in 4 % paraformaldehyde (PFA) at RT and washed with PBS three times for 15 min each. This was followed by a 1-hour incubation in blocking solution containing 0.4 % bovine serum albumin (BSA), 0.1 % Triton X-100 and 0.02 % normal donkey serum (Invitrogen) in PBS. Primary antibodies were incubated with the cultures overnight at 4 °C. After three washing steps in blocking solution for 20 min each, primaries were detected by incubation for 2 hours at RT with fluorescently conjugated secondary antibodies raised in donkey (Chemicon, Temecula, CA). To delineate the plasma membrane of cells, we labelled them with the carbocyanine dye DiI. The best results were obtained when DiI labelling was performed after immunocytochemistry. DIV20 cultures were fixed for 20 min in 4 % PFA at RT and washed with PBS three times for 15 min each. This was followed by 1-hour incubation in blocking solution containing 0.4 % BSA and 0.02 % normal donkey serum (Invitrogen) in PBS. Cultures were incubated for 48 hours at 4 °C in mouse α-NeuN (Chemicon) diluted in blocking solution with 0.001 % Triton X-100 to improve antibody penetration. Higher concentrations of Triton-X resulted in deleterious effects on subsequent DiI labelling. After three washing steps in blocking solution for 20 min each, the primary was detected by incubation for 2 hours at RT with a Cy5 conjugated secondary antibody raised in donkey (Chemicon). NeuN-stained cultures were then co-labelled with one of the following primary antibodies: goat α-NaCH type Iα (Santa Cruz, sc 31451, 1:200) or rabbit α-sodium bicarbonate co-transporter (NBC) (Millipore AB3208, 1:100), which were detected by Alexa488 conjugated secondary of the appropriate species. The nuclear stain DAPI was also included. Cultures were then incubated for 2 hours at 37 °C in a working solution of DiI (about 40 μg/ml) (Molecular Probes). Cultures were washed three to five times in PBS to remove the unbound dye. In each analysis group, cultures were processed for immunocytochemistry and DiI labelling completely in parallel, and images were captured with identical light and camera settings. For analysis of single-cell and spine morphology, cultured neurons were transfected with a pEGFP plasmid using Lipofectamine 2000 (Invitrogen) according to the manufacturer instructions.

### Morphological quantification of single GFP transfected neurons

To avoid sampling bias, we measured all GFP transfected neurons with neurites that were clearly visible and not obscured by other cells. When the processes of a neuron extended beyond the field of view, several images were taken and stitched together. Neurons were identified by expression of neuronal nuclear antigen (NeuN) (after DIV5) or basic morphology (before DIV5, due to low expression levels of NeuN at early ages). Neurites were traced with NeuronJ. For spine analysis, images were acquired from DIV20 GFP-expressing neurons at high magnification using an oil immersion 100× objective (Olympus). The number and length of dendritic spines and filopodia were measured from the primary dendrites, proximal to the cell body and extending as long as they remained in focus (for segments longer than 10 μm). We did not correct for spines hidden beneath or above the dendrite; therefore, spine densities likely underestimate the true value. Filopodia were defined as dendritic protrusions that were >3 μm in length. Spines were defined as protrusions <3 μm in length, and similar to those reported previously [93], we included protrusions that emanated laterally greater than about 0.5 μm from the dendritic shaft. The observer was not blinded as the neuronal morphology of the TeA is visibly different. However, to minimize potential bias [93], a single observer scored spine densitometry for all regions. Spines were categorized as mushroom, stubby and thin based on the morphological criteria of Peters and Kaiserman-Abramof [94]. The number of dendritic spines of each type were counted using the Cell Counter plug-in in ImageJ. The spine density was calculated by dividing the total number of spines by the length of the dendrite analysed. An average spine density was obtained for each neuron analysed.

### Tissue sections

Tissue sections were used to evaluate neuronal dormancy under more physiological conditions from a total of 57 animals. This was based on our earlier pilot data indicating that juvenile tissue likely harbours a large proportion of the dormant phenotype, and the large effect size (*d* = 0.8) that would be expected with a completely functional network in mature tissue (power = 0.8). Slices (250–450 μm) were submerged in a recording chamber in oxygenated (95 % 0_2_; 5 % CO_2_) artificial cerebrospinal fluid (aCSF). Recordings were targeted to layer V of the posterior sector of the ventral TeA-ectorhinal cortex (also known as area Te2) as this is the major output layer of the TeA. The TeA region is identified as the region located dorsal to the rhinal fissure (e.g. Plates 36–44 in [95]). Frontal and parahorizontal tissue sections were prepared on a Leica vibrotome (Germany) from Sprague-Dawley and Long-Evans rats. The aCSF had a final pH and osmolality of around 7.4 and 300 mOsm respectively and contained (mM): NaCl, 110; KCl, 3.5; MgCl_2_, 1.5; NaHCO_3_, 26; CaCl_2_, 2; glucose, 10. Recordings were made at 30–33 °C. For single-cell processing, cells were fixed after recording in 4 % PFA overnight at 4 °C. Slices were then washed with PBS three times for 10 min each and incubated in blocking solution (10 % normal goat serum, 0.25 % Triton-X 100, 0.1 M phosphate buffer) for 1 hour. Slices were then incubated in streptavidin-Cy3 (1:1000; Jackson Immunoresearch, West Grove, PA) and mouse monoclonal NeuN antibody (1:500; Chemicon International) in blocking solution for 1 hour at RT. Slices were then washed with PBS three times for 10 min and incubated with secondary fluorescence-conjugated (Alexa488) anti-mouse antibody at RT for 1 hour. Finally, slices were washed in 1× PBS three times for 10 min and then mounted, dehydrated and coverslipped with mounting medium. For 4′,6-diamidino-2-phenylindole (DAPI) staining, 20–40 μm thick sections were cut and stained with DAPI (1:10^4^) for 5 min at RT followed by a PBS rinse three times for 5 min. For mouse-NeuN and rabbit-NF-200, sections were washed three times for 10 min each with PBS and incubated with blocking solution (PBS containing 10 % NGS and 0.1 % Triton X-100) for 1 hour. Sections were then incubated with primary antibody mouse NeuN (1:500) and rabbit NF-200 (1:800) overnight at 4 °C or 1 hour at RT in blocking solution. Sections were then washed three times for 10 min each in PBS and exposed for 1 hour with donkey anti-mouse cy3 (1:250) and goat anti-rabbit cy3 (1:250) at RT diluted in PBS. Sections were then washed three times for 10 min each in PBS and mounted in aqua polymount.

### Electrophysiology

The patch electrode (DC resistance; 6–8 MΩ) solution contained (mM): K-gluconate, 120; KCl, 10; HEPES, 10; Na-GTP, 0.2; Mg-ATP/Na-ATP, 4 or 110 K-gluconate, 10 HEPES, 1.0 EGTA, 20 KCL, 2 MgCl_2_, 2.0 Na-ATP, 0.25 Na-GTP, 10 di-tris-phosphocreatine, and had a pH and osmolality of around 7.3 and 285 mOsm respectively. The bicarbonate recording solution consisted of 100 mM KCl and 50 mM NaHCO_3_. The pH was adjusted with HCl and NaOH. Under light microscopy (Zeiss Axioskop Microscope; Carl Zeiss, Germany), neurons were visually selected based on their pyramidal or ovoid morphology [96] both in vitro and in situ. Bright field images of the same visual field used for immunohistochemical analysis were also captured to confirm that visually identified neurons were positive for neuronal markers (i.e. NeuN or MAP2). Biocytin was also included in the pipette for co-labelling with the neuron-specific marker NeuN in some experiments (Additional file 4: Figure S4).

### Obtaining patch-clamp recordings for detailed electrophysiological characterization

The recording configuration was obtained by releasing the positive pipette pressure and simultaneous application of a negative pipette pressure by mouth. Care was taken to minimize mechanical perturbation of the cell/soma by ensuring that only local (i.e. the area under the pipette) membrane deformation and patch rupture occurred. This process could also be confirmed visually under DIC microscopy in some instances. In cases that were not visually confirmed, the amplifier was switched into current-clamp mode to evaluate the temporal dynamics of the recording configuration and the resting voltage potential (*I* = 0). The former is an important and easy step to rule out the fast membrane charging times associated with a cell-attached configuration despite the ability of still being able to record synaptic and action potential events in cell-attached mode [41]. Additional suction, however, often resulted in intracellular aspiration and cell deformation and/or loss of the patch.

Recordings from neurons in immature cortex typically exhibit more depolarized membrane potentials (*V*_m_) (–30 to –60 mV) that can be maintained throughout the recording session [97]. Indeed, in both cell phenotypes observed in the present study these typical membrane potentials were obtained following the patching procedure and could be maintained throughout the recording period. For spiking cells, there was an immediate polarization in *V*_m_ that remained stable for the duration of the recording that is typical of patch-clamp recordings [97]. The dormant phenotype, on the other hand, exhibited a different pattern; after the initial shift in *V*_m_ following the patching procedure, the *V*_m_ started to move toward and stabilized at more hyperpolarized potentials (e.g. –95.4 ± 10.6 mV; *n* = 10). However, this hyperpolarizing shift in *V*_m_ was prevented when using a bicarbonate pipette buffer solution (–32.7 ± 6.8 mV, *n* = 7) that would help prevent a CO_2_ gradient-dependent acidification at the membrane interfacial region [98]. Indeed, when we acidified the bicarbonate solution to effectively reduce free bicarbonate, we observed a strong effect in the opposite direction to that observed under increased free bicarbonate conditions: significantly decreased conductance (Additional file 7: Figure S7) and further *V*_m_ polarization (–138.5 ± 6.5 mV; *n* = 8; *F*_(2,22)_ = 32.09 *p* < 0.0001 and all three conditions are significantly different from each other, Tukey’s post hoc test *p* ≤ 0.01) that moved away from the chloride reversal towards the calculated Na-K-ATPase reversal based on the estimated free energy of ATP hydrolysis [99].

### Confocal microscopy

Immunoreactivity and DiI labelling were examined with an Olympus confocal FV300 laser scanning system and BX50 upright microscope using a 60× UplanApo 1.20 NA water immersion objective. Images were captured in sequential mode in double labelling experiments. Z-stack scans were taken with 1-μm optical sections. Fifteen images were captured from each coverslip by translating the stage in a systematic and unbiased fashion. Optimal light, amplifier and camera settings were defined for each of the fluorochromes used and kept consistent for all images captured. To quantify peri-plasmalemmal channel/transporter expression with confocal microscopy, we analysed the XY plane of the NeuN-positive DiI-labelled cell that was closest to the coverslip (i.e. the first Z plane where the ventral DiI signal becomes evident). We chose to analyse this flat basal part of the cellular membrane because (1) it could be identified objectively, and (2) it provided a larger surface area to analyse than the top or side slices due to their curvature. These regions of interest were defined using ImageJ (NIH, W. Rasband) and their signal intensity was measured.

### Electrical micro-stimulation

Transmembrane potentials are based on field strengths required for microtubule translocation [47]. We found that extracellular electrical stimulation on the order of 10^1^–10^2^ V/cm helped promote decoupling (*n* = 5). Extracellular electric fields can be related to transmembrane potential by a first approximation by the equation Δ*V*_m_ = –(3/2)**E***a*cosθ, where **E** represents the extracellular electric field strength, *a* is the cell radius and θ equals the polar angle [100]. This allowed us to localize stimulation to the cell we are recording from to see if we could artificially disrupt the decoupled state. This assumes that (1) the electrically insulated region is roughly spherical, (2) it has a high membrane resistance and (3) it lacks voltage-dependent changes in conductance, all of which are defining characteristics of the decoupled phenotype under standard conditions (see Results). For example, an extracellular electric field of 5 × 10^1^ V/cm, sufficient for microtubule translocation [47], would be expected to generate a maximum regional transmembrane around –150 to –200 mV under the same recording conditions as extracellular stimulation (Δ*V* + *V*_soma_). Thus, stimulation consisted of sustained repetitive short (e.g. 1 ms) duration hyperpolarizing pulses 1–10 Hz, or several series of longer duration pulses on the order of 10^1^ seconds. Shorter duration stimulation often required even greater intensity stimulation, and the process was facilitated with incremental increases in stimulation intensity following each series of stimulations.

### FM1-43 imaging

The membrane impermeable amphipathic dye FM1-43 was included in the pipette for intracellular cytosolic structural labelling. To normalize signals from different experiments, FM fluorescence measurements were determined by subtracting the background signal from the mean fluorescence signal in the somatic region. Fluorescence images were analysed using ImageJ (NIH) software. Fluorescence images were captured on-line with a Nikon CCD camera attached to an Axioscope 2 FS-mot Carl Zeiss microscope.

### Additional control experiments

There are four clear lines of evidence confirming that the decoupled phenotype is observed under a whole-cell recording configuration. First, the soma can be labelled when different membrane impermeable dyes are included in the pipette (e.g. see Fig. 3). Second, the *V*_m_ can be changed by using different pipette solutions (see above). Third, pipette dialysis, the hallmark of a whole-cell recording configuration, with hypotonic or hypertonic pipette solution leads to cell shrinkage or cell swelling/dimpling respectively (Additional file 8: Figure S8). Finally, and most notably, despite a lack of recordable cationic current, anionic current can be measured when a bicarbonate pipette solution is used and can be blocked by bath application of anionic channel blockers (Additional file 7: Figure S7). Importantly, this confirms current measurement at the level of the outer plasmalemma.

### Dialysis experiments

For the tonicity experiments, strong changes in tonicity are needed to provide the necessary driving force to override the endogenous compensatory processes and allow visualization of a response [101]. Thus, for the hypotonic condition, the standard pipette solution (285 mOsm) was diluted by adding deionized water (e.g. 1.5 ml of deionized water to 2 ml of pipette solution). For the hypertonic condition, approximately 280 mM of magnesium chloride was added to the standard pipette solution. Note that the chloride counterion likely has little effect on intracellular tonicity as it is one of the most prevalent ions involved in endogenous intracellular osmoregulation [101].

### Analysis

Signal acquisition and analysis was accomplished using the Multi-clamp 700A or Axopatch 200B and DIGIDATA 1322A 16-bit data acquisition system (Molecular Devices). Data was typically low-pass filtered at 2–8 kHz and digitized at ≥10 kHz. Intrinsic properties were determined according to methods previously described [30, 102, 103]. Data are expressed as mean ± SEM, and the statistical test is noted when used. *N* represents cells unless otherwise specified.

## Additional files

Additional file 1: Figure S1. Dormant pyramidal cells lack significant rectifying currents. Typical recordings (*inset*) obtained under voltage clamp from a dormant (*grey*) and a spiking pyramidal cell (*purple*). The former is characterized by a general absence of voltage-dependent current and displays an essentially linear current-voltage relationship. Graph represents summary data obtained from 15 cells (*n* = 8 dormant and *n* = 5 spiking). Cells were voltage clamped at –60 mV. (PDF 604 kb) Additional file 2: Figure S2. Recordable currents from TeA dendrites. A: Differential interference contrast (DIC) microscopy images of the same pyramidal neuron illustrating recordings targeted to a dendrite (*left*) and to that of the soma (*right*). Scale bar = 20 μm. B: Corresponding current traces from somatic (*red*) and dendritic (*black*) recordings in response to a voltage ramp from –120 to +50 mV. The corresponding traces from panel A are shown in *purple* and *blue* for the dendrite and soma respectively for the same cell example. Note that a total of four traces have been shown for each group (i.e. soma and dendrite). C–D: Summary data demonstrating significantly lower membrane conductance (*G*) at both hyperpolarized (–100 mV; *t*_(5)_ = 3.18 Welch-corrected *p* = 0.024) and depolarized (+50 mV; *t*_(5)_ = 3.29 Welch-corrected *p* = 0.022) potentials from somatic (*n* = 11) compared to dendritic (*n* = 6) recordings. **p* < 0.05. (PDF 184 kb) Additional file 3: Figure S3. BDNF can stimulate TeA maturation and somato-dendritic coupling. A: DIV20 cultures immunocytochemically labelled with the dendritic-associated marker MAP2 under control (TeA) and BDNF treatment (TeA + BDNF) on DIV6 (50 ng/ml). Scale bar = 50 μm. B: Summary data showing the density of MAP2 positive dendrites in DIV12–20 cultures between control and BDNF treatment (*t*_(12)_ = 5.28 *p* = 0.0002; *n* = 7 cultures). C: Single-cell GFP-labelled neurons under control (*left*) and BDNF-treated (*right*) conditions. TeA neurons were transfected with GFP plasmid on DIV12 and visualized on DIV13. Scale bar = 20 μm. D: Summary data of TeA neurite length between control and BDNF conditions (control *n* = 34 and BDNF-treated *n* = 29; *t*_(61)_ = 7.31 *p* < 0.0001). E: Summary data of the proportion of dormant neurons. The first two bars (Juvenile and Mature) represent re-plotted data from intact tissue at different ages representing the total proportion of neurons that exhibit the dormant phenotype (P10-28 vs. P29-49; 78 % (*n* = 157) in mature vs. 41 % (*n* = 94) in juvenile; *p* < 0.0001, Fisher’s exact test), while the last two bars (TeA and TeA + BDNF) represent TeA cultures without (*n* = 16; DIV12–15) and with BDNF treatment (*n* = 15; DIV13–14, 75 % without and 33 % with; *p* = 0.032, Fisher’s exact test) respectively. F: *Left*; a DIC image of the dimensions of a TeA pyramidal cell indicating the long and short somatic axes. Note that dendrites could always be observed under DIC microscopy. With a lack of any significant voltage-gated conductances (e.g. Additional file 1: Figure S1), the specific membrane capacitance is expected to be ≈ 0.4–0.75 μF/cm^2^ [104–106]. Given the approximate dimensions of the soma, we can thus estimate the theoretical somatic capacitance (diameter equal to the average of the long and short axes). *Inset*; the theoretical and measured *C*_m_ values for this cell using a value of 0.5 μF/cm^2^. *Middle* and *right*; images represent Alexa488 (40 μM) fluorescence images of a small capacitance pyramidal cell characteristic of the TeA and that of a spiking cell following BDNF application with confirmed aqueous dye diffusing into dendrites respectively. Scale bar = 20 μm. Colours correspond to summary data on the *far right* (*purple n* = 13; *blue n* = 9; *orange n* = 10; *F*_(2,29)_ = 119.51, *p* < 0.0001; ANOVA and Games-Howell post-test, BDNF *p* < 0.0001). (PDF 232 kb) Additional file 4: Figure S4. Dormant cells are neuronal in nature. A: A biocytin-filled dormant neuron (*left*) and NeuN co-staining of the same section (*right*) illustrating the nuclear region of the same cell (*dotted circle*). Scale bar = 25 μm. B: Summary data (*t*_(4)_ = 6.83 *p* = 0.0024 *n* = 5; one-sample *t* test) of NeuN immunoreactivity. To make individual fluorescence measurements comparable, we normalized the ’co-labelled’ NeuN signals to the highest NeuN signals (i.e. pixel density) obtained from the same sections. Co-labelled cells generally had a less dense and more diffuse NeuN staining pattern. C: To ensure that diffuse NeuN staining was not due to recording conditions, NeuN signals were analysed for a population of TeA neurons and normalized for comparison in the same way as in panel B: a high percentage of TeA neurons have moderate to low levels of NeuN staining. ***p* < 0.01. (PDF 673 kb) Additional file 5: Figure S5. Lack of age-dependent increase in cell number in TeA. A: Representative images of juvenile and mature tissue sections illustrating a comparable pattern of DAPI (*blue*) and NeuN (*green*) staining. Neurofilament-200 (*red*) was also included here to illustrate the surrounding neuropil. Note that the intensity of DAPI staining appears, in general, weaker and more dispersed in older animals. Scale bar = 50 μm. B–C: Summarized cell count data based on DAPI (B) and NeuN (C) staining normalized for comparison. Note that there does not appear to be an increase in cell number for both the total number of cells and NeuN positive cells between juvenile (P19; *n* = 3 animals) and mature (P57; *n* = 3 animals) animals using both counting methods (*t*_(4)_ = 1.45 *p* = 0.22 for DAPI and *t*_(4)_ = 1.69 *p* = 0.17 for NeuN). These results are consistent with the general notion that the postnatal neocortex does not appear to produce more neurons [51]. (PDF 695 kb) Additional file 6: Figure S6. TTX-sensitive spikes in spiking neurons from mature tissue. A: Representative current-clamp step-protocol traces from a spiking neuron under pre (Control) and post-tetrodotoxin (sodium channel blocker) application (TTX). The step protocol consisted of an initial –200 pA step followed by eight 50 pA steps (750 ms in duration) from an initial *V*_m_ of –70 mV. B: Summarized data (*n* = 5) on the effect of TTX (1–0.1 μM) on the number of spikes per second (normalized) under control and TTX conditions in spiking neurons from tissue slices around 1 month old (*t*_(4)_ = –29.7; *p* < 0.0001). (PDF 200 kb) Additional file 7: Figure S7. Presence of anionic but not cationic membrane current. A: Representative current traces (*left*) for both low free bicarbonate (*red*; *n* = 15; pH 3.5–4; 50 mM NaHCO_3_) and high free bicarbonate ion (*black*; *n* = 14; pH 8–8.5; 50 mM NaHCO_3_) internal pipette recording conditions are shown in A. Cells were held at –60 mV and a voltage command ramp from –120 to +50 mV was applied. (*Right*) Group data illustrating a significant larger slope conductance (*G*) under alkaline conditions (*t*_(13)_ = 3.80 Welch-correction *p* = 0.0022 on square rooted-transformed data to minimize distribution skew). Importantly, under these conditions, cells still failed to demonstrate action potential and rectifying currents under both ramp or step protocols. B: Given the lack of any observable current under acidic recording conditions, the membrane impermeable amphipathic dye FM1-43 (≈40 μM) was included in the pipette (*n* = 10). Scale bar = 20 μm. C: Current traces (*left*) from the same cell under high free bicarbonate internal recording conditions before (*black*) and after (*orange*) bath application of the anionic (chloride and electrogenic NBC co-transporter) channel blockers SITS and DIDS (≈1 mM). Cells were held at a holding potential of –60 mV and a voltage command ramp from –160 to +80 mV was applied. (*Right*) Summary data showing that the high free bicarbonate internal recording condition membrane current is sensitive to both SITS and/or DIDS. Bath application of SITS and/or DIDS led to a significant decrease in recordable current (Wilcoxon signed-rank test *W* = –36.0, *p* = 0.0039 *n* = 8). When stratified into individual or combined drug application, similar findings were observed (*t*_(3)_ = 5.60 *p* = 0.011 one-sample *t* test and *t*_(3)_ = 33.38 *p* < 0.0001 one-sample *t* test for either and both blockers respectively), with almost 90 % of the recordable current being blocked with SITS and DIDS application. **p* < 0.025; ***p* < 0.01. (PDF 1103 kb) Additional file 8: Figure S8. Optical changes driven by the imposed pipette tonicity. A–B: A schematic illustration of the experiments to confirm a whole-cell configuration based on the characteristic feature that, under whole-cell conditions, the properties of the pipette solution are imposed to the cytosol through dialysis. Here a hypotonic pipette solution was used to provide a driving force for the movement of ions from the cell interior to the pipette during dialysis (*red arrow*; panel A). Following dialysis (e.g. >3 min) the interior of the cell becomes like the pipette solution (i.e. hypotonic), thus leading to a crenation-like effect in cell shape as water moves out of the cell (panel A). Conversely, using a hypertonic pipette solution will result in the opposite effect; ions move into the cell and ultimately lead to cell swelling membrane dimpling (panel B). C: Representative examples of these types of responses, which were quite robust occurring in 100 % of cells tested. Under these conditions cells still failed to demonstrate action potential and rectifying currents. D: This response can be partially quantified using an ImageJ profile analysis where the *grey* value represents regions of high contrast (e.g. edges) from the DIC image region of interest (*orange line*; the *coloured arrows* correspond to the same spatial locations as in panel C). It should be mentioned that unlike the XY planes of the cell which are associated with the coverslip, changes could also be observed in the Z plane but were difficult to quantify. The colors correspond to the initial condition (*green*) and the dialyzed condition (*orange*) for both panels C and D. E: The summarized data represent the proportional change in cross-sectional distance of dormant cells for both the hypertonic (*light grey*; *n* = 6; *t*_(5)_ = 4.28, one-sampled *t* test *p* = 0.0079) and hypotonic (*dark grey*; *n* = 5; *t*_(4)_ = 10.51, one-sampled *t* test *p* = 0.0005) conditions. Note that volume increases non-linearly with distance. * denotes *p* < 0.05. Scale bar = 5 μm. (PDF 148 kb) Additional file 9: Fig. Data Source File. (XLS 97 kb)

## References

[1] Hishida R, Kamatani D, Kitaura H, Kudoh M, Shibuki K (2007). Functional local connections with differential activity-dependence and critical periods surrounding the primary auditory cortex in rat cerebral slices. Neuroimage.

[2] Luna B, Garver KE, Urban TA, Lazar NA, Sweeney JA (2004). Maturation of cognitive processes from late childhood to adulthood. Child Dev.

[3] Scherf KS, Behrmann M, Humphreys K, Luna B (2007). Visual category-selectivity for faces, places and objects emerges along different developmental trajectories. Dev Sci.

[4] Geier CF, Garver K, Terwilliger R, Luna B (2009). Development of working memory maintenance. J Neurophysiol.

[5] Golarai G, Ghahremani DG, Whitfield-Gabrieli S, Reiss A, Eberhardt JL, Gabrieli JD (2007). Differential development of high-level visual cortex correlates with category-specific recognition memory. Nat Neurosci.

[6] Elman JL (1993). Learning and development in neural networks: the importance of starting small. Cognition.

[7] Quartz SR (1999). The constructivist brain. Trends Cogn Sci.

[8] Guillery RW (2005). Is postnatal neocortical maturation hierarchical?. Trends Neurosci.

[9] Knudsen EI, Heckman JJ, Cameron JL, Shonkoff JP (2006). Economic, neurobiological, and behavioral perspectives on building America’s future workforce. Proc Natl Acad Sci U S A.

[10] Grill-Spector K, Golarai G, Gabrieli J (2008). Developmental neuroimaging of the human ventral visual cortex. Trends Cogn Sci.

[11] Knudsen EI (2004). Sensitive periods in the development of the brain and behavior. J Cogn Neurosci.

[12] Shaw P, Eckstrand K, Sharp W, Blumenthal J, Lerch JP, Greenstein D (2007). Attention-deficit/hyperactivity disorder is characterized by a delay in cortical maturation. Proc Natl Acad Sci U S A.

[13] Sowell ER, Peterson BS, Thompson PM, Welcome SE, Henkenius AL, Toga AW (2003). Mapping cortical change across the human life span. Nat Neurosci.

[14] Rakic P, Bourgeois JP, Eckenhoff MF, Zecevic N, Goldman-Rakic PS (1986). Concurrent overproduction of synapses in diverse regions of the primate cerebral cortex. Science.

[15] Quartz SR, Sejnowski TJ (1997). The neural basis of cognitive development: a constructivist manifesto. Behav Brain Sci.

[16] Zhang LI, Poo MM (2001). Electrical activity and development of neural circuits. Nat Neurosci.

[17] Dennis EL, Jahanshad N, McMahon KL, de Zubicaray GI, Martin NG, Hickie IB (2013). Development of brain structural connectivity between ages 12 and 30: a 4-Tesla diffusion imaging study in 439 adolescents and adults. Neuroimage..

[18] Khundrakpam BS, Reid A, Brauer J, Carbonell F, Lewis J, Ameis S (2013). Developmental changes in organization of structural brain networks. Cereb Cortex.

[19] Cohen Kadosh K, Johnson MH, Dick F, Cohen Kadosh R, Blakemore SJ (2013). Effects of age, task performance, and structural brain development on face processing. Cereb Cortex.

[20] Chomiak T, Karnik V, Block E, Hu B (2010). Altering the trajectory of early postnatal cortical development can lead to structural and behavioural features of autism. BMC Neurosci..

[21] Chomiak T, Hung H, Cihal A, Dhaliwal J, Baghdadwala MI, Agata D (2014). Auditory-cued sensorimotor task reveals disengagement deficits in rats exposed to the autism-associated teratogen valproic acid. Neuroscience..

[22] Kossel AH, Williams CV, Schweizer M, Kater SB (1997). Afferent innervation influences the development of dendritic branches and spines via both activity-dependent and non-activity-dependent mechanisms. J Neurosci.

[23] Frotscher M, Drakew A, Heimrich B (2000). Role of afferent innervation and neuronal activity in dendritic development and spine maturation of fascia dentata granule cells. Cereb Cortex.

[24] Balasubramaniyan V, de Haas AH, Bakels R, Koper A, Boddeke HW, Copray JC (2004). Functionally deficient neuronal differentiation of mouse embryonic neural stem cells in vitro. Neurosci Res.

[25] Zhang ZN, Freitas BC, Qian H, Lux J, Acab A, Trujillo CA (2016). Layered hydrogels accelerate iPSC-derived neuronal maturation and reveal migration defects caused by MeCP2 dysfunction. Proc Natl Acad Sci U S A.

[26] Berry M, Rogers AW (1965). The migration of neuroblasts in the developing cerebral cortex. J Anat.

[27] Hotulainen P, Hoogenraad CC (2010). Actin in dendritic spines: connecting dynamics to function. J Cell Biol.

[28] Spitzer NC (2006). Electrical activity in early neuronal development. Nature.

[29] Moyer J, Brown T, Walz W, Boulton AA, Baker GB (2002). Patch-clamp techniques to brain slices. Patch-clamp analysis: advanced techniques.

[30] Picken Bahrey HL, Moody WJ (2003). Early development of voltage-gated ion currents and firing properties in neurons of the mouse cerebral cortex. J Neurophysiol.

[31] Carleton A, Petreanu LT, Lansford R, Alvarez-Buylla A, Lledo PM (2003). Becoming a new neuron in the adult olfactory bulb. Nat Neurosci.

[32] Esposito MS, Piatti VC, Laplagne DA, Morgenstern NA, Ferrari CC, Pitossi FJ (2005). Neuronal differentiation in the adult hippocampus recapitulates embryonic development. J Neurosci.

[33] Cambray S, Arber C, Little G, Dougalis AG, de Paola V, Ungless MA (2012). Activin induces cortical interneuron identity and differentiation in embryonic stem cell-derived telencephalic neural precursors. Nat Commun..

[34] Gong B, Rhodes KJ, Bekele-Arcuri Z, Trimmer JS (1999). Type I and type II Na(+) channel alpha-subunit polypeptides exhibit distinct spatial and temporal patterning, and association with auxiliary subunits in rat brain. J Comp Neurol.

[35] Douglas RM, Schmitt BM, Xia Y, Bevensee MO, Biemesderfer D, Boron WF (2001). Sodium-hydrogen exchangers and sodium-bicarbonate co-transporters: ontogeny of protein expression in the rat brain. Neuroscience.

[36] Goodman CS, Spitzer NC (1981). The development of electrical properties of identified neurones in grasshopper embryos. J Physiol..

[37] Horton AC, Ehlers MD (2004). Secretory trafficking in neuronal dendrites. Nat Cell Biol.

[38] Williams SR (2004). Spatial compartmentalization and functional impact of conductance in pyramidal neurons. Nat Neurosci.

[39] Migliore M, Shepherd GM (2002). Emerging rules for the distributions of active dendritic conductances. Nat Rev Neurosci.

[40] Bekkers JM, Hausser M (2007). Targeted dendrotomy reveals active and passive contributions of the dendritic tree to synaptic integration and neuronal output. Proc Natl Acad Sci U S A.

[41] Perkins KL (2006). Cell-attached voltage-clamp and current-clamp recording and stimulation techniques in brain slices. J Neurosci Methods.

[42] Horton AC, Ehlers MD (2003). Dual modes of endoplasmic reticulum-to-Golgi transport in dendrites revealed by live-cell imaging. J Neurosci.

[43] Anderson RL, Jobling P, Gibbins IL (2001). Development of electrophysiological and morphological diversity in autonomic neurons. J Neurophysiol.

[44] Franceschetti S, Sancini G, Panzica F, Radici C, Avanzini G (1998). Postnatal differentiation of firing properties and morphological characteristics in layer V pyramidal neurons of the sensorimotor cortex. Neuroscience.

[45] Luby-Phelps K (2000). Cytoarchitecture and physical properties of cytoplasm: volume, viscosity, diffusion, intracellular surface area. Int Rev Cytol..

[46] Seelig J, Macdonald PM, Scherer PG (1987). Phospholipid head groups as sensors of electric charge in membranes. Biochemistry.

[47] Kim T, Kao MT, Hasselbrink EF, Meyhofer E (2007). Active alignment of microtubules with electric fields. Nano Lett.

[48] Patel N, Poo MM (1982). Orientation of neurite growth by extracellular electric fields. J Neurosci.

[49] Yuste R, Gutnick MJ, Saar D, Delaney KR, Tank DW (1994). Ca2+ accumulations in dendrites of neocortical pyramidal neurons: an apical band and evidence for two functional compartments. Neuron.

[50] Sengupta P (2013). The laboratory rat: relating its age with human’s. Int J Prev Med.

[51] Rakic P (2006). Neuroscience. No more cortical neurons for you. Science.

[52] Gonchar Y, Burkhalter A (1997). Three distinct families of GABAergic neurons in rat visual cortex. Cereb Cortex.

[53] Chomiak T, Peters S, Hu B (2008). Functional architecture and spike timing properties of corticofugal projections from rat ventral temporal cortex. J Neurophysiol.

[54] Torre ER, Steward O (1992). Demonstration of local protein synthesis within dendrites using a new cell culture system that permits the isolation of living axons and dendrites from their cell bodies. J Neurosci.

[55] Eberwine J, Miyashiro K, Kacharmina JE, Job C (2001). Local translation of classes of mRNAs that are targeted to neuronal dendrites. Proc Natl Acad Sci U S A.

[56] Holt CE, Schuman EM (2013). The central dogma decentralized: new perspectives on RNA function and local translation in neurons. Neuron.

[57] Safronov BV, Wolff M, Vogel W (1999). Axonal expression of sodium channels in rat spinal neurones during postnatal development. J Physiol.

[58] Ye B, Zhang Y, Song W, Younger SH, Jan LY, Jan YN (2007). Growing dendrites and axons differ in their reliance on the secretory pathway. Cell.

[59] Kalil K, Li L, Hutchins BI (2011). Signaling mechanisms in cortical axon growth, guidance, and branching. Front Neuroanat..

[60] Hefti BJ, Smith PH (2000). Anatomy, physiology, and synaptic responses of rat layer V auditory cortical cells and effects of intracellular GABA(A) blockade. J Neurophysiol.

[61] Connors BW, Gutnick MJ (1990). Intrinsic firing patterns of diverse neocortical neurons. Trends Neurosci.

[62] Sills JB, Connors BW, Burwell RD (2012). Electrophysiological and morphological properties of neurons in layer 5 of the rat postrhinal cortex. Hippocampus.

[63] Mainen ZF, Sejnowski TJ (1996). Influence of dendritic structure on firing pattern in model neocortical neurons. Nature.

[64] Song AH, Wang D, Chen G, Li Y, Luo J, Duan S (2009). A selective filter for cytoplasmic transport at the axon initial segment. Cell.

[65] Moody WJ, Bosma MM (2005). Ion channel development, spontaneous activity, and activity-dependent development in nerve and muscle cells. Physiol Rev.

[66] D’Angelo E, De Filippi G, Rossi P, Taglietti V (1997). Synaptic activation of Ca2+ action potentials in immature rat cerebellar granule cells in situ. J Neurophysiol.

[67] Duch C, Levine RB (2000). Remodeling of membrane properties and dendritic architecture accompanies the postembryonic conversion of a slow into a fast motoneuron. J Neurosci.

[68] Bernier PJ, Parent A (1998). Bcl-2 protein as a marker of neuronal immaturity in postnatal primate brain. J Neurosci.

[69] Sia Y, Bourne JA (2008). The rat temporal association cortical area 2 (Te2) comprises two subdivisions that are visually responsive and develop independently. Neuroscience.

[70] Arimatsu Y, Ishida M, Takiguchi-Hayashi K, Uratani Y (1999). Cerebral cortical specification by early potential restriction of progenitor cells and later phenotype control of postmitotic neurons. Development.

[71] McAllister AK, Lo DC, Katz LC (1995). Neurotrophins regulate dendritic growth in developing visual cortex. Neuron.

[72] Maisonpierre PC, Belluscio L, Friedman B, Alderson RF, Wiegand SJ, Furth ME (1990). NT-3, BDNF, and NGF in the developing rat nervous system: parallel as well as reciprocal patterns of expression. Neuron.

[73] Horch HW, Katz LC (2002). BDNF release from single cells elicits local dendritic growth in nearby neurons. Nat Neurosci.

[74] Cohen MS, Bas Orth C, Kim HJ, Jeon NL, Jaffrey SR (2011). Neurotrophin-mediated dendrite-to-nucleus signaling revealed by microfluidic compartmentalization of dendrites. Proc Natl Acad Sci U S A.

[75] Fanger GR, Jones JR, Maue RA (1995). Differential regulation of neuronal sodium channel expression by endogenous and exogenous tyrosine kinase receptors expressed in rat pheochromocytoma cells. J Neurosci.

[76] Matsuda N, Lu H, Fukata Y, Noritake J, Gao H, Mukherjee S (2009). Differential activity-dependent secretion of brain-derived neurotrophic factor from axon and dendrite. J Neurosci.

[77] Kuczewski N, Porcher C, Lessmann V, Medina I, Gaiarsa JL (2009). Activity-dependent dendritic release of BDNF and biological consequences. Mol Neurobiol.

[78] Yu SP, Yeh CH, Sensi SL, Gwag BJ, Canzoniero LM, Farhangrazi ZS (1997). Mediation of neuronal apoptosis by enhancement of outward potassium current. Science.

[79] Payne JA, Rivera C, Voipio J, Kaila K (2003). Cation-chloride co-transporters in neuronal communication, development and trauma. Trends Neurosci.

[80] Smith RL, Clayton GH, Wilcox CL, Escudero KW, Staley KJ (1995). Differential expression of an inwardly rectifying chloride conductance in rat brain neurons: a potential mechanism for cell-specific modulation of postsynaptic inhibition. J Neurosci.

[81] Franciolini F, Nonner W (1987). Anion and cation permeability of a chloride channel in rat hippocampal neurons. J Gen Physiol.

[82] Naya Y, Yoshida M, Miyashita Y (2001). Backward spreading of memory-retrieval signal in the primate temporal cortex. Science.

[83] Komura Y, Tamura R, Uwano T, Nishijo H, Kaga K, Ono T (2001). Retrospective and prospective coding for predicted reward in the sensory thalamus. Nature.

[84] Mooney DM, Zhang L, Basile C, Senatorov VV, Ngsee J, Omar A (2004). Distinct forms of cholinergic modulation in parallel thalamic sensory pathways. Proc Natl Acad Sci U S A.

[85] Grosso A, Cambiaghi M, Renna A, Milano L, Roberto Merlo G, Sacco T (2015). The higher order auditory cortex is involved in the assignment of affective value to sensory stimuli. Nat Commun..

[86] Sacco T, Sacchetti B (2010). Role of secondary sensory cortices in emotional memory storage and retrieval in rats. Science.

[87] Zielinski BA, Prigge MB, Nielsen JA, Froehlich AL, Abildskov TJ, Anderson JS (2013). Longitudinal changes in cortical thickness in autism and typical development. Brain.

[88] Chomiak T, Hu B (2013). Alterations of neocortical development and maturation in autism: Insight from valproic acid exposure and animal models of autism. Neurotoxicol Teratol..

[89] Chomiak T, Turner N, Hu B (2013). What we have learned about autism spectrum disorder from valproic acid. Patholog Res Int..

[90] Paxinos G, Tork I, Tecott LH (1991). Atlas of the developing rat brain.

[91] Shi CJ, Cassell MD (1997). Cortical, thalamic, and amygdaloid projections of rat temporal cortex. J Comp Neurol.

[92] Shi C, Davis M (2001). Visual pathways involved in fear conditioning measured with fear-potentiated startle: behavioral and anatomic studies. J Neurosci.

[93] Holtmaat A, Bonhoeffer T, Chow DK, Chuckowree J, De Paola V, Hofer SB (2009). Long-term, high-resolution imaging in the mouse neocortex through a chronic cranial window. Nat Protoc.

[94] Peters A, Kaiserman-Abramof IR (1970). The small pyramidal neuron of the rat cerebral cortex. The perikaryon, dendrites and spines. Am J Anat.

[95] Paxinos G, Watson C (1998). The rat brain: in stereotaxic coordinates.

[96] DeFelipe J, Farinas I (1992). The pyramidal neuron of the cerebral cortex: morphological and chemical characteristics of the synaptic inputs. Prog Neurobiol.

[97] Tyzio R, Ivanov A, Bernard C, Holmes GL, Ben-Ari Y, Khazipov R (2003). Membrane potential of CA3 hippocampal pyramidal cells during postnatal development. J Neurophysiol.

[98] Genz AK, v Engelhardt W, Busche R (1999). Maintenance and regulation of the pH microclimate at the luminal surface of the distal colon of guinea-pig. J Physiol.

[99] Trotier D, Doving KB (1996). Direct influence of the sodium pump on the membrane potential of vomeronasal chemoreceptor neurones in frog. J Physiol.

[100] Gross D, Loew LM, Webb WW (1986). Optical imaging of cell membrane potential changes induced by applied electric fields. Biophys J.

[101] O’Neill WC (1999). Physiological significance of volume-regulatory transporters. Am J Physiol.

[102] Cruikshank SJ, Lewis TJ, Connors BW (2007). Synaptic basis for intense thalamocortical activation of feedforward inhibitory cells in neocortex. Nat Neurosci.

[103] Wu RL, Butler DM, Barish ME (1998). Potassium current development and its linkage to membrane expansion during growth of cultured embryonic mouse hippocampal neurons: sensitivity to inhibitors of phosphatidylinositol 3-kinase and other protein kinases. J Neurosci.

[104] White SH (1970). A study of lipid bilayer membrane stability using precise measurements of specific capacitance. Biophys J.

[105] Tien HT (1988). Bilayer lipid membrane-based electrochemical biosensors. J Clin Lab Anal..

[106] Shepherd GM, Zigmond MJ, Bloom FE, Landis SC, Roberts JL, Squire LR (1999). Electrotonic properties of axons and dendrites. Fundamental neuroscience.

