# Supplementary figures and images for: Somato-dendritic decoupling as a novel mechanism for protracted cortical maturation
Source: BMC Biol. 2016 Jun 21;14:48. doi: 10.1186/s12915-016-0270-5 (PMC4916537; doi:10.1186/s12915-016-0270-5)

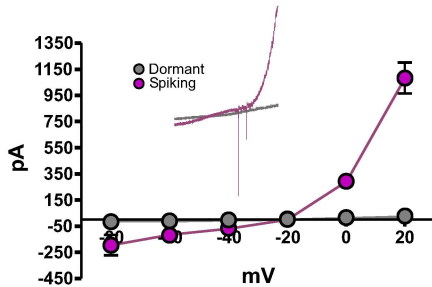

Supplement: Additional file 1: Figure S1. — Dormant pyramidal cells lack significant rectifying currents. Typical recordings (inset) obtained under voltage clamp from a dormant (grey) and a spiking pyramidal cell (purple). The former is characterized by a general absence of voltage-dependent current and displays an essentially linear current-voltage relationship. Graph represents summary data obtained from 15 cells (n = 8 dormant and n = 5 spiking). Cells were voltage clamped at –60 mV. (PDF 604 kb) [file 12915_2016_270_MOESM1_ESM.pdf]

**A**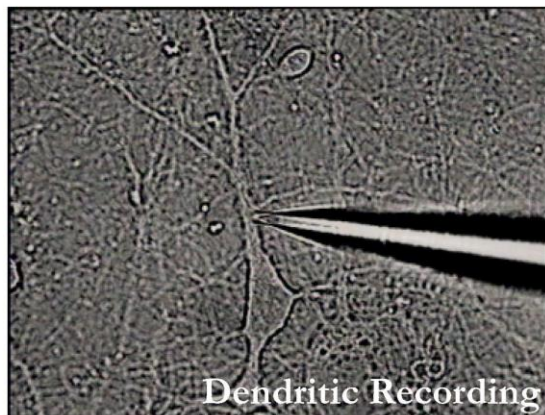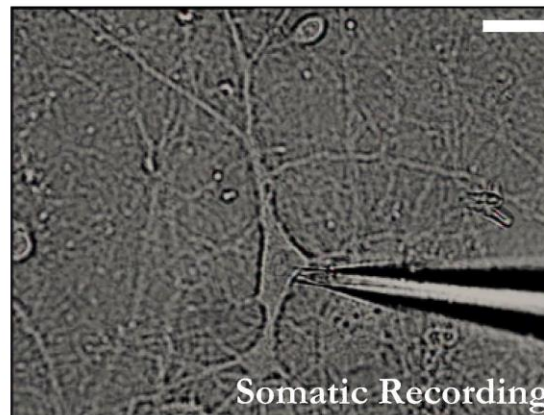**B**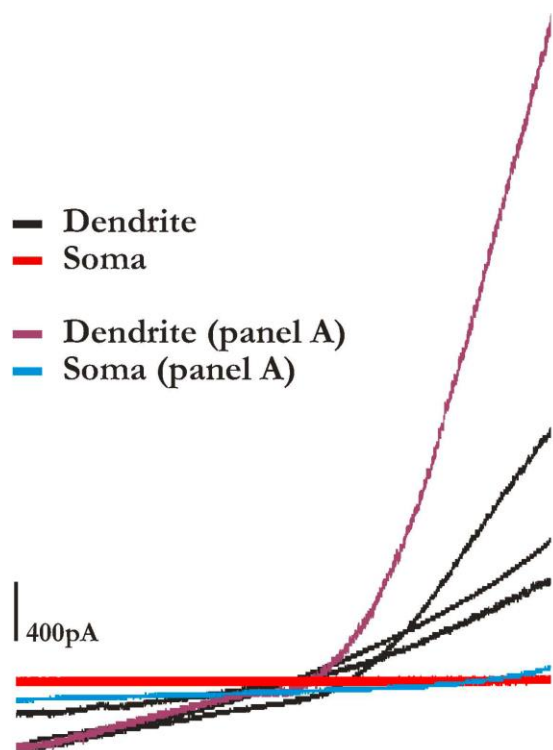**C**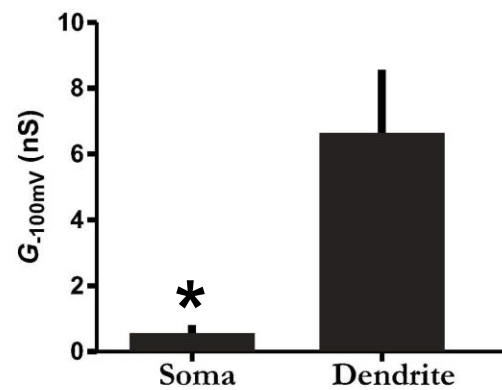**D**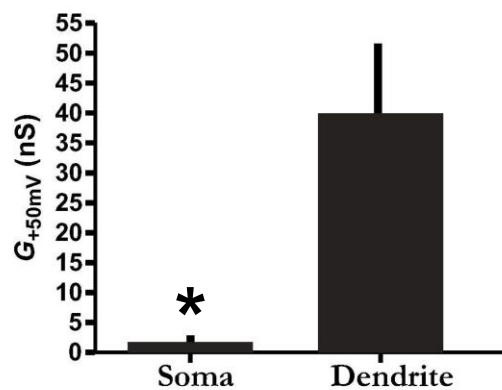

Supplement: Additional file 2: Figure S2. — Recordable currents from TeA dendrites. A: Differential interference contrast (DIC) microscopy images of the same pyramidal neuron illustrating recordings targeted to a dendrite (left) and to that of the soma (right). Scale bar = 20 μm. B: Corresponding current traces from somatic (red) and dendritic (black) recordings in response to a voltage ramp from –120 to +50 mV. The corresponding traces from panel A are shown in purple and blue for the dendrite and soma respectively for the same cell example. Note that a total of four traces have been shown for each group (i.e. soma and dendrite). C–D: Summary data demonstrating significantly lower membrane conductance (G) at both hyperpolarized (–100 mV; t (5) = 3.18 Welch-corrected p = 0.024) and depolarized (+50 mV; t (5) = 3.29 Welch-corrected p = 0.022) potentials from somatic (n = 11) compared to dendritic (n = 6) recordings. *p < 0.05. (PDF 184 kb) [file 12915_2016_270_MOESM2_ESM.pdf]

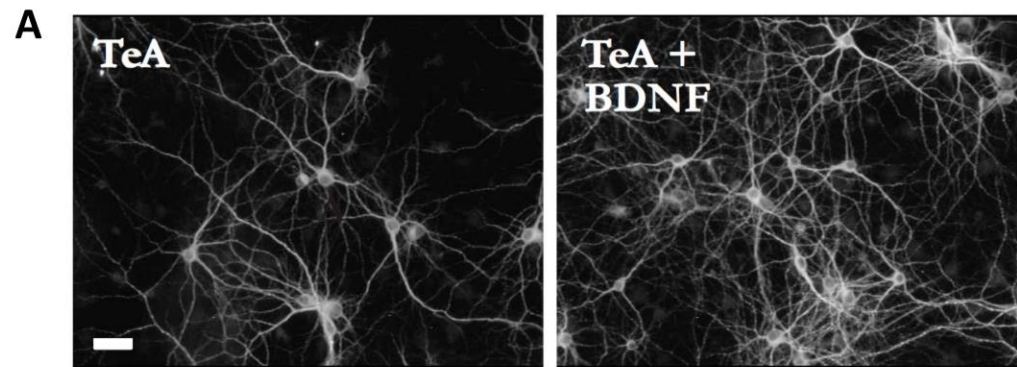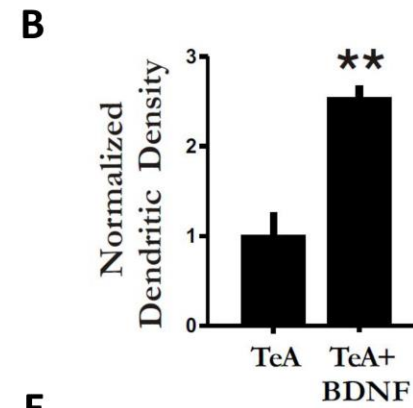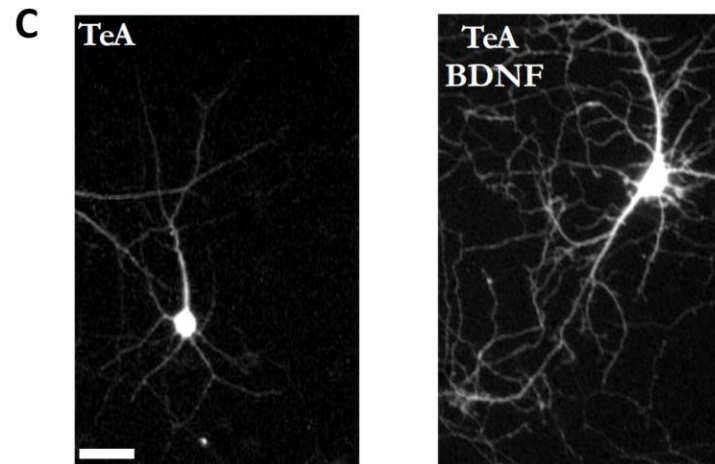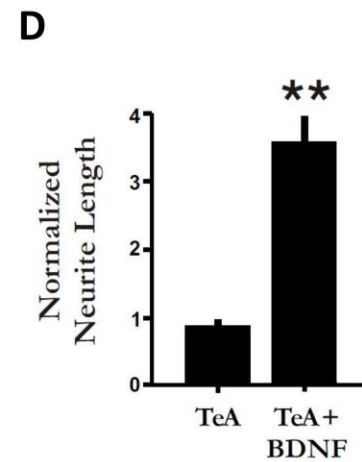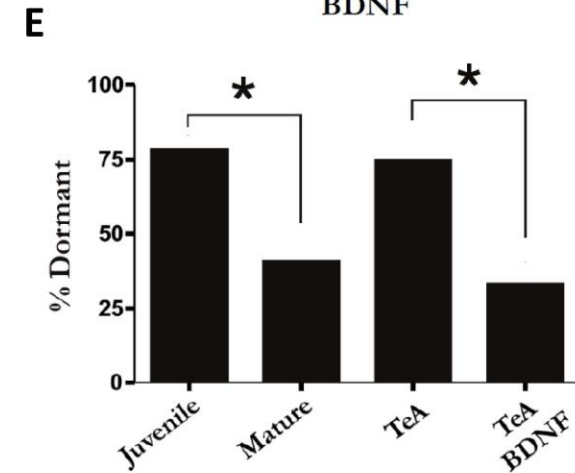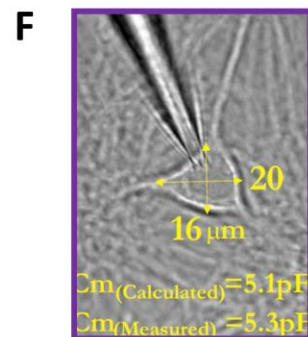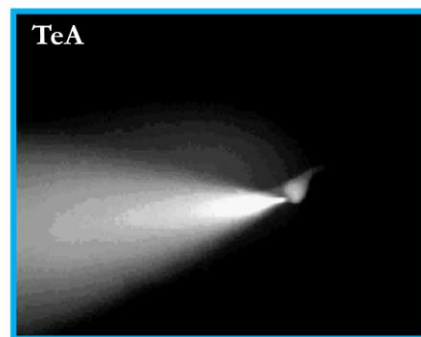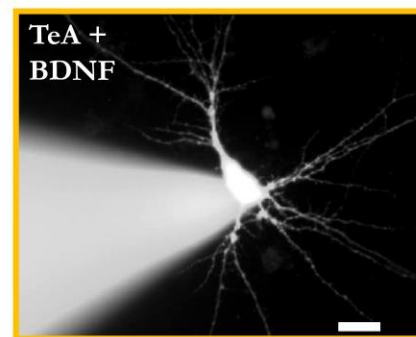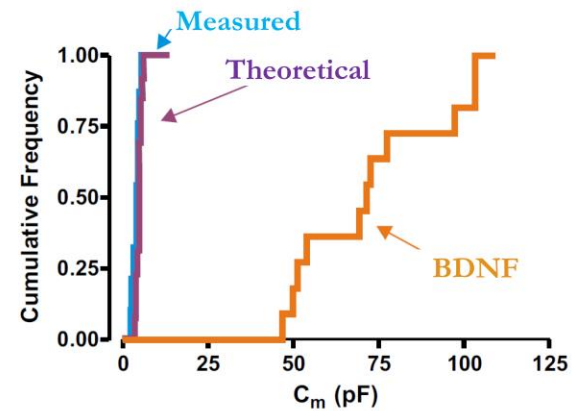

Supplement: Additional file 3: Figure S3. — BDNF can stimulate TeA maturation and somato-dendritic coupling. A: DIV20 cultures immunocytochemically labelled with the dendritic-associated marker MAP2 under control (TeA) and BDNF treatment (TeA + BDNF) on DIV6 (50 ng/ml). Scale bar = 50 μm. B: Summary data showing the density of MAP2 positive dendrites in DIV12–20 cultures between control and BDNF treatment (t (12) = 5.28 p = 0.0002; n = 7 cultures). C: Single-cell GFP-labelled neurons under control (left) and BDNF-treated (right) conditions. TeA neurons were transfected with GFP plasmid on DIV12 and visualized on DIV13. Scale bar = 20 μm. D: Summary data of TeA neurite length between control and BDNF conditions (control n = 34 and BDNF-treated n = 29; t (61) = 7.31 p < 0.0001). E: Summary data of the proportion of dormant neurons. The first two bars (Juvenile and Mature) represent re-plotted data from intact tissue at different ages representing the total proportion of neurons that exhibit the dormant phenotype (P10-28 vs. P29-49; 78 % (n = 157) in mature vs. 41 % (n = 94) in juvenile; p < 0.0001, Fisher’s exact test), while the last two bars (TeA and TeA + BDNF) represent TeA cultures without (n = 16; DIV12–15) and with BDNF treatment (n = 15; DIV13–14, 75 % without and 33 % with; p = 0.032, Fisher’s exact test) respectively. F: Left; a DIC image of the dimensions of a TeA pyramidal cell indicating the long and short somatic axes. Note that dendrites could always be observed under DIC microscopy. With a lack of any significant voltage-gated conductances (e.g. Additional file 1: Figure S1), the specific membrane capacitance is expected to be ≈ 0.4–0.75 μF/cm2 [104–106]. Given the approximate dimensions of the soma, we can thus estimate the theoretical somatic capacitance (diameter equal to the average of the long and short axes). Inset; the theoretical and measured C m values for this cell using a value of 0.5 μF/cm2. Middle and right; images represent Alexa488 (40 μM) fluorescence images of a small capacitanc [file 12915_2016_270_MOESM3_ESM.pdf]

**A**

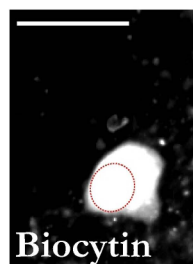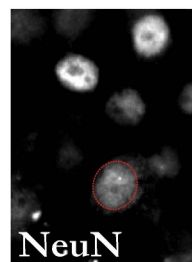

**B**

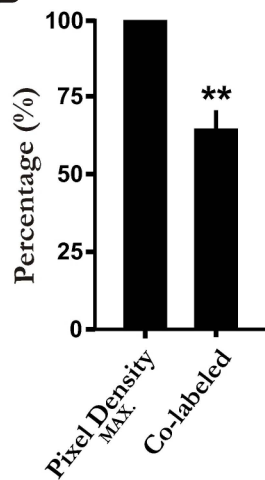

**C**

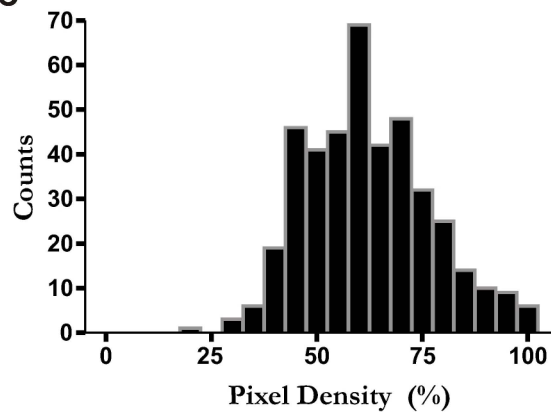

Supplement: Additional file 4: Figure S4. — Dormant cells are neuronal in nature. A: A biocytin-filled dormant neuron (left) and NeuN co-staining of the same section (right) illustrating the nuclear region of the same cell (dotted circle). Scale bar = 25 μm. B: Summary data (t (4) = 6.83 p = 0.0024 n = 5; one-sample t test) of NeuN immunoreactivity. To make individual fluorescence measurements comparable, we normalized the ’co-labelled’ NeuN signals to the highest NeuN signals (i.e. pixel density) obtained from the same sections. Co-labelled cells generally had a less dense and more diffuse NeuN staining pattern. C: To ensure that diffuse NeuN staining was not due to recording conditions, NeuN signals were analysed for a population of TeA neurons and normalized for comparison in the same way as in panel B: a high percentage of TeA neurons have moderate to low levels of NeuN staining. **p < 0.01. (PDF 673 kb) [file 12915_2016_270_MOESM4_ESM.pdf]

**A**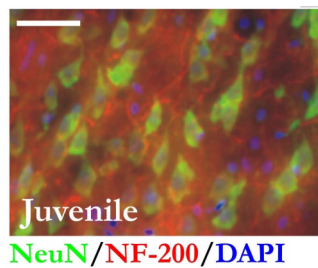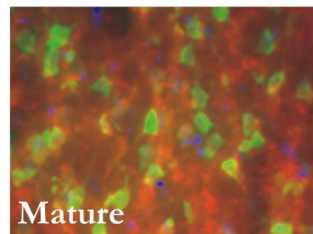**B**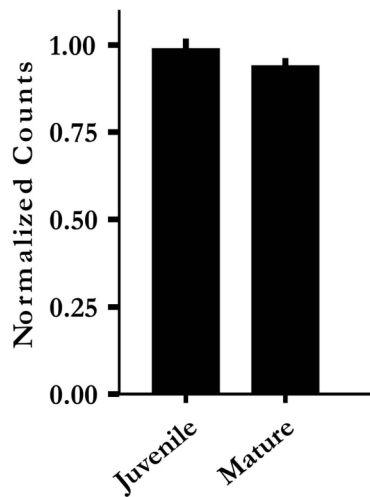**C**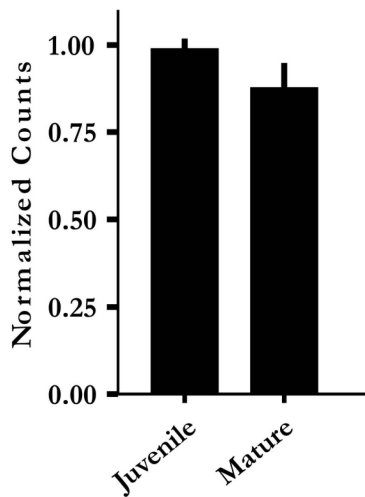

Supplement: Additional file 5: Figure S5. — Lack of age-dependent increase in cell number in TeA. A: Representative images of juvenile and mature tissue sections illustrating a comparable pattern of DAPI (blue) and NeuN (green) staining. Neurofilament-200 (red) was also included here to illustrate the surrounding neuropil. Note that the intensity of DAPI staining appears, in general, weaker and more dispersed in older animals. Scale bar = 50 μm. B–C: Summarized cell count data based on DAPI (B) and NeuN (C) staining normalized for comparison. Note that there does not appear to be an increase in cell number for both the total number of cells and NeuN positive cells between juvenile (P19; n = 3 animals) and mature (P57; n = 3 animals) animals using both counting methods (t (4) = 1.45 p = 0.22 for DAPI and t (4) = 1.69 p = 0.17 for NeuN). These results are consistent with the general notion that the postnatal neocortex does not appear to produce more neurons [51]. (PDF 695 kb) [file 12915_2016_270_MOESM5_ESM.pdf]

**A**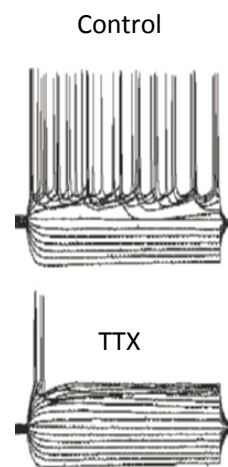**B**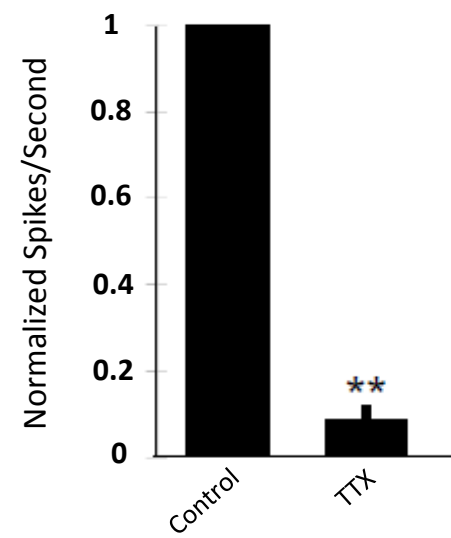

Supplement: Additional file 6: Figure S6. — TTX-sensitive spikes in spiking neurons from mature tissue. A: Representative current-clamp step-protocol traces from a spiking neuron under pre (Control) and post-tetrodotoxin (sodium channel blocker) application (TTX). The step protocol consisted of an initial –200 pA step followed by eight 50 pA steps (750 ms in duration) from an initial V m of –70 mV. B: Summarized data (n = 5) on the effect of TTX (1–0.1 μM) on the number of spikes per second (normalized) under control and TTX conditions in spiking neurons from tissue slices around 1 month old (t (4) = –29.7; p < 0.0001). (PDF 200 kb) [file 12915_2016_270_MOESM6_ESM.pdf]

**A**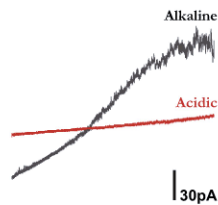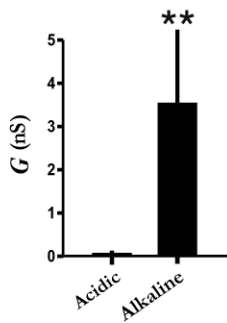**B**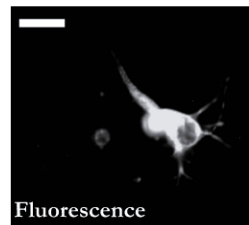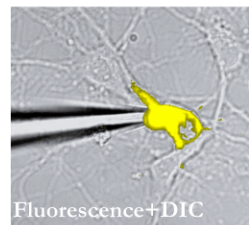**C**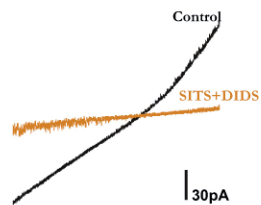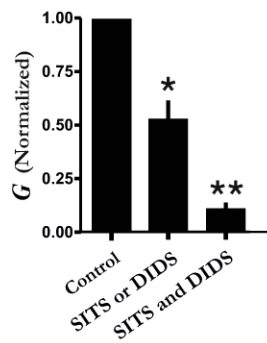

Supplement: Additional file 7: Figure S7. — Presence of anionic but not cationic membrane current. A: Representative current traces (left) for both low free bicarbonate (red; n = 15; pH 3.5–4; 50 mM NaHCO3) and high free bicarbonate ion (black; n = 14; pH 8–8.5; 50 mM NaHCO3) internal pipette recording conditions are shown in A. Cells were held at –60 mV and a voltage command ramp from –120 to +50 mV was applied. (Right) Group data illustrating a significant larger slope conductance (G) under alkaline conditions (t (13) = 3.80 Welch-correction p = 0.0022 on square rooted-transformed data to minimize distribution skew). Importantly, under these conditions, cells still failed to demonstrate action potential and rectifying currents under both ramp or step protocols. B: Given the lack of any observable current under acidic recording conditions, the membrane impermeable amphipathic dye FM1-43 (≈40 μM) was included in the pipette (n = 10). Scale bar = 20 μm. C: Current traces (left) from the same cell under high free bicarbonate internal recording conditions before (black) and after (orange) bath application of the anionic (chloride and electrogenic NBC co-transporter) channel blockers SITS and DIDS (≈1 mM). Cells were held at a holding potential of –60 mV and a voltage command ramp from –160 to +80 mV was applied. (Right) Summary data showing that the high free bicarbonate internal recording condition membrane current is sensitive to both SITS and/or DIDS. Bath application of SITS and/or DIDS led to a significant decrease in recordable current (Wilcoxon signed-rank test W = –36.0, p = 0.0039 n = 8). When stratified into individual or combined drug application, similar findings were observed (t (3) = 5.60 p = 0.011 one-sample t test and t (3) = 33.38 p < 0.0001 one-sample t test for either and both blockers respectively), with almost 90 % of the recordable current being blocked with SITS and DIDS application. *p < 0.025; **p < 0.01. (PDF 1103 kb) [file 12915_2016_270_MOESM7_ESM.pdf]

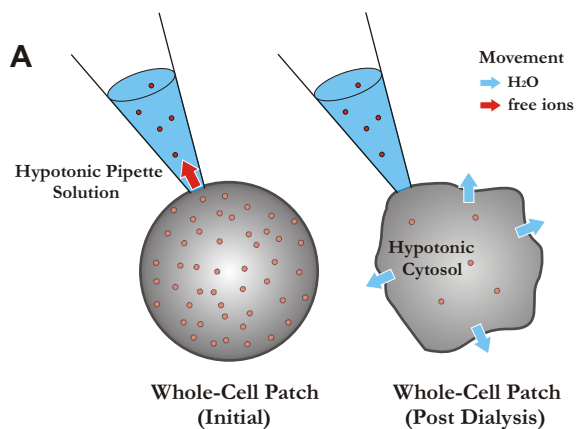

**C**

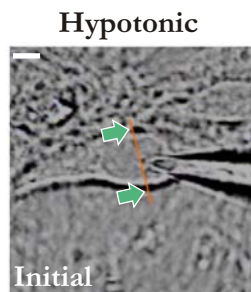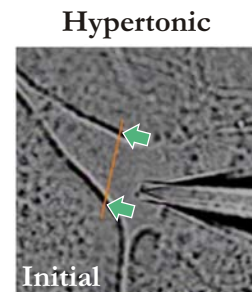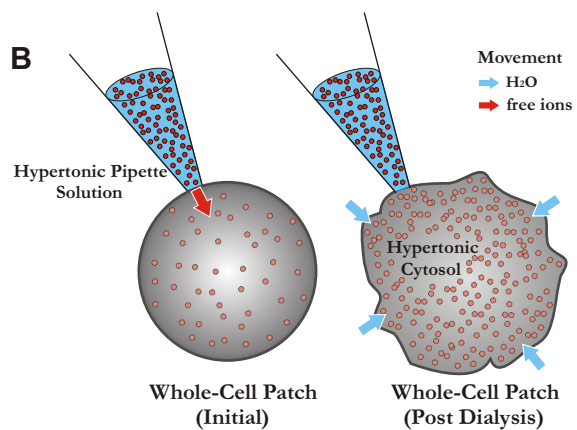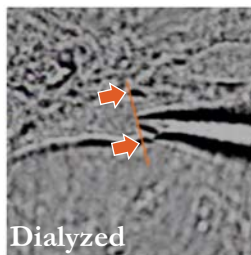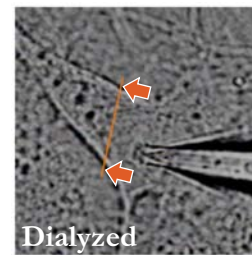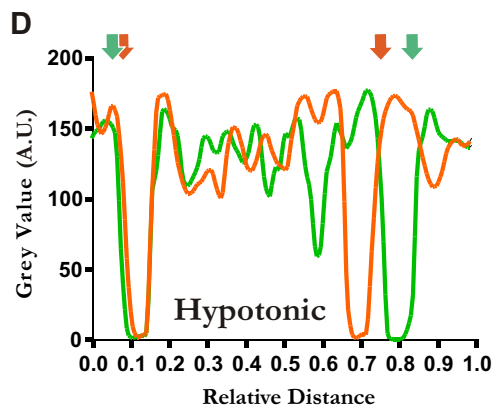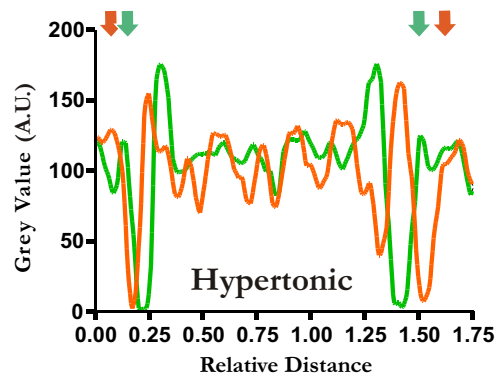

**E**

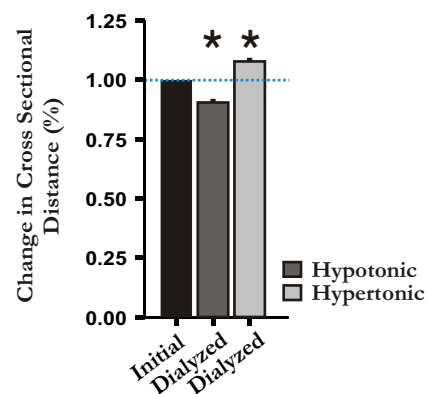

Supplement: Additional file 8: Figure S8. — Optical changes driven by the imposed pipette tonicity. A–B: A schematic illustration of the experiments to confirm a whole-cell configuration based on the characteristic feature that, under whole-cell conditions, the properties of the pipette solution are imposed to the cytosol through dialysis. Here a hypotonic pipette solution was used to provide a driving force for the movement of ions from the cell interior to the pipette during dialysis (red arrow; panel A). Following dialysis (e.g. >3 min) the interior of the cell becomes like the pipette solution (i.e. hypotonic), thus leading to a crenation-like effect in cell shape as water moves out of the cell (panel A). Conversely, using a hypertonic pipette solution will result in the opposite effect; ions move into the cell and ultimately lead to cell swelling membrane dimpling (panel B). C: Representative examples of these types of responses, which were quite robust occurring in 100 % of cells tested. Under these conditions cells still failed to demonstrate action potential and rectifying currents. D: This response can be partially quantified using an ImageJ profile analysis where the grey value represents regions of high contrast (e.g. edges) from the DIC image region of interest (orange line; the coloured arrows correspond to the same spatial locations as in panel C). It should be mentioned that unlike the XY planes of the cell which are associated with the coverslip, changes could also be observed in the Z plane but were difficult to quantify. The colors correspond to the initial condition (green) and the dialyzed condition (orange) for both panels C and D. E: The summarized data represent the proportional change in cross-sectional distance of dormant cells for both the hypertonic (light grey; n = 6; t (5) = 4.28, one-sampled t test p = 0.0079) and hypotonic (dark grey; n = 5; t (4) = 10.51, one-sampled t test p = 0.0005) conditions. Note that volume increases non-linearly with distance. * denotes p < 0.05. Scale [file 12915_2016_270_MOESM8_ESM.pdf]
